# Pain intensity, physical function, and depressive symptoms associated with discontinuing long‐term opioid therapy in older adults with Alzheimer's disease and related dementias

**DOI:** 10.1002/alz.13489

**Published:** 2023-10-19

**Authors:** Yu‐Jung Jenny Wei, Almut G. Winterstein, Siegfried Schmidt, Roger B. Fillingim, Michael J. Daniels, Laurence Solberg, Steven T. DeKosky

**Affiliations:** ^1^ Division of Outcomes and Translational Sciences College of Pharmacy The Ohio State University Columbus Ohio USA; ^2^ Department of Pharmaceutical Outcomes and Policy College of Pharmacy University of Florida Gainesville Florida USA; ^3^ Center for Drug Evaluation and Safety University of Florida Gainesville Florida USA; ^4^ Department of Epidemiology Colleges of Medicine and Public Health & Health Professions University of Florida Gainesville Florida USA; ^5^ Department of Community Health and Family Medicine College of Medicine University of Florida Gainesville Florida USA; ^6^ Pain Research and Intervention Center of Excellence University of Florida Gainesville Florida USA; ^7^ Department of Statistics College of Liberal Arts and Sciences University of Florida Gainesville Florida USA; ^8^ North Florida/South Georgia Veterans Health System Malcom Randall Department of Veterans Affairs Medical Center Geriatrics Research, Education, Clinical Center (GRECC) Gainesville Florida USA; ^9^ Department of Neurology McKnight Brain Institute University of Florida Gainesville Florida USA

**Keywords:** Alzheimer's disease and related dementias, clinical outcomes, long‐term opioid therapy, Medicare

## Abstract

**INTRODUCTION:**

Limited evidence exists on the associations of discontinuing versus continuing long‐term opioid therapy (LTOT) with pain intensity, physical function, and depression among patients with Alzheimer's disease and related dementias (ADRD).

**METHODS:**

A cohort study among 138,059 older residents with mild‐to‐moderate ADRD and receipt of LTOT was conducted using a 100% Medicare nursing home sample. Discontinuation of LTOT was defined as no opioid refills for ≥ 60 days. Outcomes were worsening pain, physical function, and depression from baseline to quarterly assessments during 1‐ and 2‐year follow‐ups.

**RESULTS:**

The adjusted odds of worsening pain and depressive symptoms were 29% and 5% lower at the 1‐year follow‐up and 35% and 9% lower at the 2‐year follow‐up for residents who discontinued versus continued LTOT, with no difference in physical function.

**DISCUSSION:**

Discontinuing LTOT was associated with lower short‐ and long‐term worsening pain and depressive symptoms than continuing LTOT among older residents with ADRD.

**Highlights:**

- Discontinuing long‐term opioid therapy (LTOT) was associated with lower short‐ and long‐term worsening pain.
- Discontinuing LTOT was related to lower short‐ and long‐term worsening depression.
- Discontinuing LTOT was not associated with short‐ and long‐term physical function.

## 1 INTRODUCTION

Pain is common among older adults with Alzheimer's disease and related dementias (ADRD), with three in four affected individuals reporting regular pain.^1^ Opioid therapy has been a major component of comprehensive pain treatment for older adults with chronic pain given that non‐opioid therapies are linked to renal and liver toxicity^2^ and gastrointestinal bleeding^3^ and that adjuvant analgesic tricyclic antidepressants may cause serious sedation, leading to falls among older adults.^4^ Those pain treatments are recommended to be avoided or used cautiously among older individuals,^5^ which leaves opioids as an available option for pain control. Use of prescription opioids for chronic pain management has tripled in US community‐dwelling older patients with ADRD (15% in 2005 to 45% in 2015).^6^, ^7^ In nursing homes (NHs), nearly half (48%) of residents with ADRD in 2015 received prescription opioids in the year after receiving a chronic pain diagnosis.^6^ Among opioid users with ADRD, about half (43%–53%) received long‐term opioid therapy (LTOT; i.e., ≥ 3 months) between 2011 and 2017.^6^

With the apparent increasing use of opioids in ADRD, a key clinical question is whether opioids are safe when used long term in this fast‐growing population. Current guidelines, such as the Centers for Disease Control and Prevention's *CDC Guideline for Prescribing Opioids for Chronic Pain—United States, 2016* and its revision in 2022, recommend appropriately tapering and discontinuing LTOT for patients without cancer or not receiving palliative care, including individuals with ADRD, when risks outweigh the benefits of LTOT.^8^, ^9^ Those clinical guidelines are based on evidence adapted from data derived from adults who are not older or older adults with healthy cognition and are generated on concerns of LTOT‐related serious harms, including respiratory depression, opioid use disorder, and fatal and non‐fatal overdose.^8^, ^9^ Yet these adverse events, largely driven by opioid misuse, or high dosages (e.g., ≥ 90 morphine milligrams equivalent [MME]/day), are rare in ADRD.^6^, ^10^ For example, the estimated prevalence of opioid use disorder in 2018 is < 1 case per 1000 beneficiaries among older patients with ADRD (based on our preliminary data) compared to 15.7 per 1000 beneficiaries among US older adults.^11^ Nevertheless, patients with ADRD and receipt of LTOT have been disproportionately affected, with an increased rate of LTOT treatment being discontinued, compared to their counterparts without ADRD between 2011 and 2018.^12^

Today, limited evidence exists regarding the short‐ and long‐term clinical effects of discontinuing LTOT on the health outcomes of patients with ADRD.^13^ We aimed to examine the associations of discontinuation versus continuation of LTOT with three key clinical outcomes—pain intensity, physical function, and depression status—among older Medicare NH residents with ADRD.

## 2 METHODS

### 2.1 Study source and design

This retrospective cohort study analyzed data from a 100% NH sample linked to Medicare claims and Minimum Data Set (MDS) 3.0 assessment from January 1, 2010, to December 31, 2020. Medicare claims data contain fee‐for‐service beneficiaries’ medical billing records for Parts A (inpatient), B (office‐based visits), and D (prescription drugs) and beneficiary‐level information on demographic characteristics, enrollment status, and date of death.^14^ The MDS 3.0 assessment is required at admission, regular intervals during a Medicare‐covered short‐term stay, and quarterly thereafter.^15^ We used quarterly MDS 3.0 assessments to measure key outcomes and important covariates, such as ADRD severity, body mass index, and use of as‐needed (PRN) pain medications among NH residents. The Ohio State University's Institutional Review Board approved this study. This study followed the Strengthening the Reporting of Observational Studies in Epidemiology (STROBE) reporting guideline.

**RESEARCH IN CONTEXT**

1. **Systematic review**: We identified no population‐based study that examined the clinical outcomes of discontinuing versus continuing long‐term opioid therapy (LTOT) in older adults, particularly those with Alzheimer's disease and related dementias (ADRD).
2. **Interpretation**: We found patients who discontinued (vs. continued) LTOT had lower odds of worsening pain and worsening depression and had no difference in physical function at 1‐year and 2‐year follow‐ups.
3. **Future directions**: While our findings provide evidence of the clinical benefits, future investigations into the safety of discontinuation versus continuation of LTOT are warranted to provide comprehensive benefit–risk evidence among patients with ADRD.

### 2.2 Study sample

The study sample included NH residents aged > 65 years who received a diagnosis of ADRD, defined based on a Centers for Medicare & Medicaid Services–prespecified diagnostic algorithm for ADRD,^14^ and received LTOT, defined as having at least one episode of opioid treatment lasting 90 days or longer with an allowable gap of fewer than 30 days to account for delays in fills of prescription opioids,^12^ during a 12‐month NH stay. Residents entered the cohort on the 90th day of their latest LTOT episode (cohort entry), were assessed as to whether LTOT was continued or discontinued, and assigned an exposure index date (described in *Key exposure*) in 1 year after cohort entry. Residents were followed up from the assigned index date for 1 year for assessing short‐term outcomes, and a subset of residents who had 2 years of follow‐up was used for examining long‐term outcomes.

To study a population homogeneous for pain conditions, we restricted residents to individuals who had received a diagnosis of chronic pain but were without cancer, palliative, or hospice care during the study period (from 12 months before cohort entry to the end of the 1‐ or 2‐year follow‐up). Diagnosis for chronic pain has shown high accuracy (from 82.9% to 93.2% depending on pain conditions) compared to medical chart review in identifying patients with chronic pain.^16^ The list of diagnoses for chronic pain was adapted mainly from a chronic pain validation study,^16^ supplemented by other clinical studies focusing on chronic pain^17^, ^18^ and the expertise of our co‐authors (Fillingim, Schmidt, and Solberg) in pain and pain management.

**FIGURE 1:**
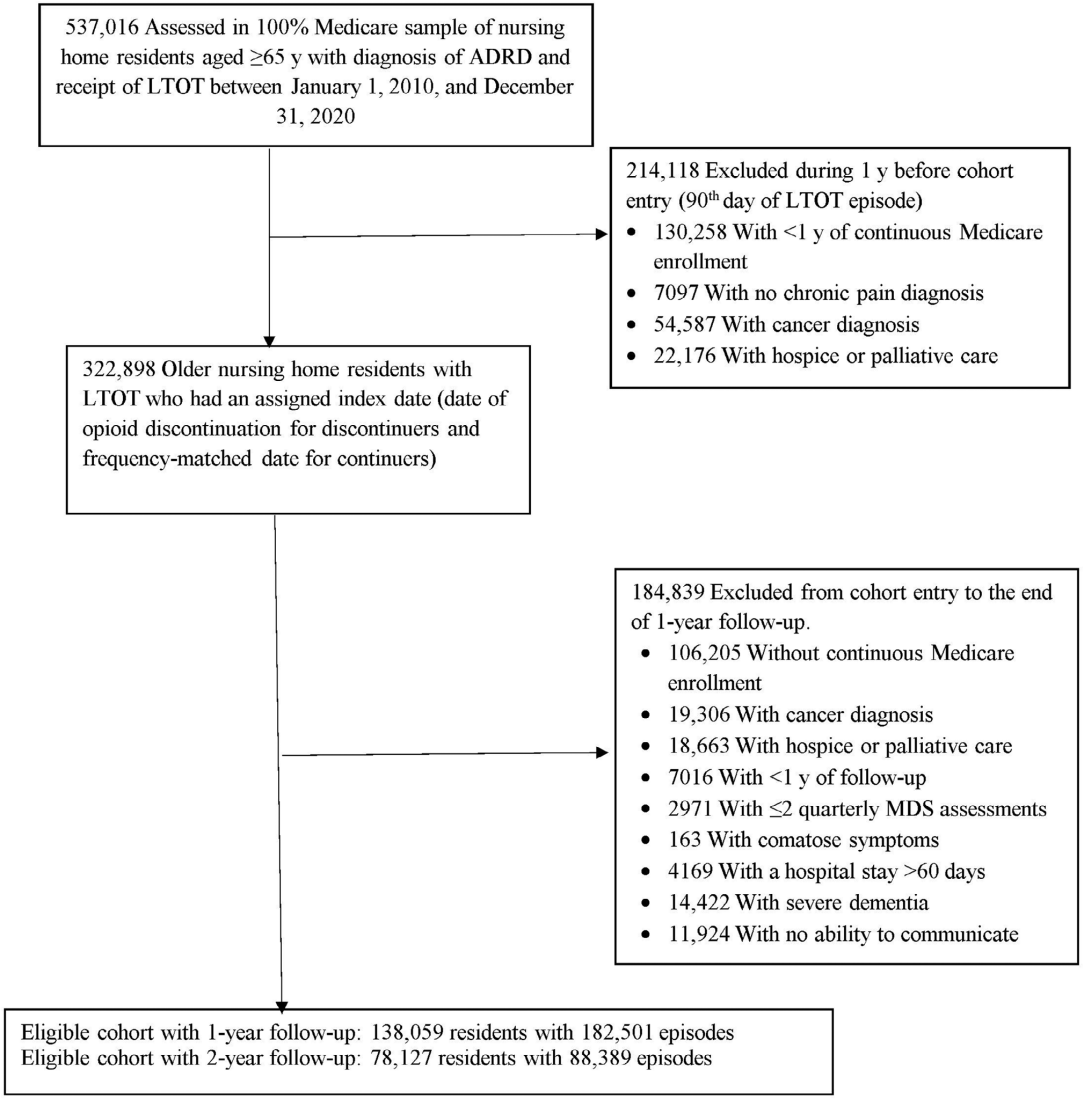
Cohort inclusion flowchart for the study sample. ADRD, Alzheimer's disease and related dementias; LTOT, long‐term opioid therapy; MDS, Minimum Data Set

Exclusion criteria included (1) no continuous Medicare enrollment, (2) presence of enrollment in Medicare Advantage plan, (3) not having two or more MDS quarterly assessments, (4) presence of comatose symptoms (documented in MDS 3.0), and (5) no continuous NH stay (determined based on admission, assessment, and discharge dates in MDS 3.0) throughout the study period. To avoid misclassification of discontinuation of LTOT (defined as a continuous gap of > 60 days) among residents with long hospital stays during which no prescription drug information was available, we further excluded residents with a hospital stay of ≥ 60 days during the period from cohort entry to the end of the follow‐up. Finally, we excluded residents with severe cognitive impairment, defined as having a Cognitive Performance Scale (CPS) score of 5 or 6^19^ or losing the ability to communicate, assessed using the quarterly MDS assessment within 6 months and closest to the index date because their pain and depression symptoms tend to be underdetected. Residents were allowed to re‐enter the cohort after the end of follow‐up if they met the eligibility criteria. Figure 1 shows the sample selection details, and Figure S1 in supporting information shows the schematic diagram of the study design. The medications of interest and diagnostic and procedure codes for conditions and services considered in the sample selection are given in Tables S1 and S2 in supporting information.

### 2.3 Key exposure

The key exposure of interest was discontinuation of LTOT measured for 1 year after cohort entry. Residents were classified as “discontinuers” if they had no opioid fills for ≥ 60 consecutive days; otherwise, as “continuers.” A continuous gap of at least 60 days was chosen on the basis that the cutoff was twice the 99th percentile of days’ supply for an opioid prescription (i.e., 30 days) dispensed by the studied residents with ADRD. For discontinuers, an index date was the date of discontinuation, defined as the date of the last prescription for opioids in addition to days’ supply. For continuers, the index date was randomly selected by frequency matching to discontinuers’ index date to ensure a similar distribution of time between cohort entry and index date between continuer and discontinuer groups. Because residents’ opioid use may change over time, we reassessed whether residents discontinued LTOT at quarterly intervals throughout follow‐up.

### 2.4 Outcome measures

The primary outcome measures were pain intensity, physical function, and depression status assessed by quarterly MDS 3.0 assessments. Pain intensity was measured by residents’ recall of worst pain in the last 5 days based on a 10‐point numerical rating scale (higher scores indicating greater pain) or a four‐level categorical verbal descriptor scale (no, mild, moderate, or severe pain).^154^ To facilitate analysis, scores of the two pain scales were combined and classified into four categories: no (0), mild (1), moderate (2), and severe (3) pain.^20^ Physical function was measured by the nine‐item (i.e., bed mobility, transfer, walking in room, walking in corridor, locomotion on unit, dressing, eating, toilet use, and personal hygiene) self‐care activities of daily living (ADLs), with each item scored from 0 (total independence) to 4 (total dependence).^21^ The total ADL score ranged from 0 to 36, with higher scores indicating worse physical function. Depression status was measured using the Patient Health Questionnaire‐9 (PHQ‐9), with each of the nine items scored from 0 (not at all) to 3 (nearly every day). The total score ranged from 0 to 27, with higher scores indicating worse depression.^22^

In each quarter of the follow‐up years, we calculated individual change in outcomes by subtracting each patient's pain, ADL, and PHQ‐9 scores from the corresponding baseline outcome extracted from a quarterly MDS assessment within 6 months before and closest to the index date. In follow‐up quarters in which at least two quarterly MDS assessments were present, we selected the latest assessment for analysis. To reflect clinical improvement for residents with ADRD who stayed in NHs for long‐term care, we dichotomized residents into two groups according to the continuous quarterly score change of each outcome: worsening outcome, defined as any increase in outcome score or remaining moderate‐to‐severe symptoms (pain score ≥ 2, ADL score ≥ 19, or PHQ‐9 score ≥ 10) from baseline to quarterly assessments; otherwise, improved outcome.

### 2.5 Study covariates

Study covariates are described in Table S3 in supporting information. Demographic characteristics include age, sex, race and ethnicity, region of the United States, receipt of low‐income subsidy, body mass index, and years of living with ADRD since the first diagnosis. Clinical conditions included a diagnosis of tobacco, alcohol, or drug use disorder, several conditions potentially affecting opioid treatment,^23^ and the total number of comorbidities. Health care use included hospitalization and emergency department visits. Clinical conditions and health care use were measured 12 months before the index date.

We measured pain management, medication use, presence of opioid‐related adverse effects, presence of dementia‐related behavioral and psychological symptoms, and dementia severity (mild or moderate) 6 months before the index date to have information more proximate to exposure status. Pain management included the use of drug or non‐drug pain management, use of PRN pain medications, receipt of medical procedures (e.g., nerve blocks) and therapies (e.g., physical and occupational therapy) for managing chronic pain, opioid dosage, use of long‐acting opioids, use of prescription non‐opioids, and use of adjuvant analgesics. Medication use included use of other central nervous system (CNS) medications, use of antidementia drugs, and polypharmacy (use of ≥ 5 dispensed generic drugs excluding opioids), potential factors associated with the outcomes of interest.^24^, ^25^, ^26^, ^27^ Using the MDS 3.0, we measured the presence of opioid‐related adverse effects (including delirium, constipation, vomiting, and dehydration), presence of behavioral and psychological symptoms (including psychotic and disruptive behaviors),^28^ and dementia severity using the CPS (range, 0–6) and the Brief Interview for Mental Status (BIMS; range 0–15).^19^ In the study sample of residents with ADRD, given that severe dementia was excluded, we accounted for baseline ADRD severity by whether residents had mild or moderate dementia.^19^ All baseline MDS‐assessed variables were extracted from the quarterly MDS assessment within 6 months and closest to the index date. Finally, to account for changes in clinical opioid prescribing due to implementation of new guidelines, regulations, and reimbursement policies during the last 10 years, we adjusted for the year of the index date.

### 2.6 Missing data

Small proportions of residents had missing data on the MDS‐assessed clinical outcomes (< 1% at baseline and during follow‐up) and MDS‐assessed covariates (< 0.3% at baseline and follow‐up). Those residents were excluded from final regression analyses. Therefore, only residents with completed MDS assessments at baseline and in any given quarter contributed to quarterly outcomes.

### 2.7 Statistical analysis

We assessed baseline covariates and baseline pain, physical function, and depressive symptoms outcomes between LTOT discontinuer and continuer groups, with a standardized mean difference (SMD) > 0.1 indicating covariate imbalance.^29^ Potential differences in baseline variables between groups were then adjusted via inverse probability of treatment weighting (IPTW), calculated as the inverse of the propensity score for LTOT discontinuers and inverse of 1 minus the propensity score for LTOT continuers. To account for confounders that changed over time (including use of adjuvant analgesics, use of PRN pain medications, use of any pain intervention, use of CNS medication, and dementia severity), we calculated the IPTW at each quarter given the previous status on discontinuation of LTOT and previous values of the time‐varying confounders and baseline covariates.^30^ Each IPTW weight was truncated at the 1st and 99th percentiles to reduce the influence of outliers on estimates.

We used a separate logistic model with IPTW to model the adjusted proportion of individuals with a worsening outcome from baseline to each quarterly follow‐up assessment among residents who discontinued versus continued LTOT. A generalized estimating equation was used for estimation to account for within‐individual correlation. We reported the odds ratios between exposure groups and their 95% confidence intervals (CIs) for each quarter and each outcome. To examine whether the associations between LTOT discontinuation and clinical outcomes changed over time, we built a separate logistic generalized estimating equation model for each outcome that included the exposure, quarterly time, and interaction of both variables, with adjustment of baseline variables via IPTW and the time‐varying confounders as covariates in the models.

We performed five sensitivity analyses to assess the robustness of the estimates: (1) modeling discontinuation of LTOT defined as no opioid fills for at least 90 consecutive days; (2) stratifying analyses according to baseline mild or moderate dementia severity; (3) stratifying analyses according to baseline pain, physical function, and depressive symptoms; (4) stratifying analyses according to types of pain conditions (classified as musculoskeletal pain, neuropathic pain, and idiopathic pain) at baseline; and (5) stratifying analyses by race and ethnicity. All analyses were conducted using SAS, version 9.4 (SAS Institute Inc.). Statistical significance was set as *P* < 0.05, and all tests were two‐sided.

## 3 RESULTS

We identified 138,059 Medicare NH residents with ADRD and receipt of LTOT who had at least 1 year of follow‐up (mean [standard deviation (SD)] age, 83.0 [11.0] years; 111,931 [81.1%] female; 26,128 [18.9%] male; 114,239 [82.7%] White; 15,388 [11.1%] Black; and 8432 [6.1%] other race and ethnicity), consisting of 182,501 resident episodes from 2010 to 2020 (Table 1). In this sample, we identified a subset of 78,127 residents who had at least 2 years of follow‐up (mean [SD] age, 82.4 [8.6] years; 64,055 [82.0%] female), consisting of 88,389 patient episodes (Table S4 in supporting information).

**TABLE 1:** Clinical and demographic characteristics of nursing home residents with ADRD and receipt of LTOT, overall and by continuation versus discontinuation of LTOT.

	Number (%) of residents^a^			SDiff^b^	
Characteristic	Overall sample (*n* = 138,059)	LTOT continuers (*n* = 137,309)^c^	LTOT discontinuers (*n* = 45,192)^c^	Before IPTW	After IPTW
**Age, years**					
Mean (SD)	83.0 (11.0)	83.3 (8.7)	82.2 (8.7)	0.131	0.000
65–74	27,906 (20.2)	25,933 (18.9)	10,228 (22.6)		
75–84	44,734 (32.4)	44,543 (32.4)	15,431 (34.1)		
≥85	65,419 (47.4)	66,833 (48.7)	19,533 (43.2)		
**Female**	111,931 (81.1)	113,371 (82.6)	35,527 (78.6)	0.100	0.006
**Male**	26,128 (18.9)	23,938 (17.4)	9665 (21.4)		
**Race and ethnicity**				0.152	0.000
White	114,239 (82.7)	115,489 (84.1)	35,252 (78.0)		
Black	15,388 (11.1)	14,410 (10.5)	6336 (14.0)		
Other^d^	8432 (6.1)	7410 (5.4)	3604 (8.0)		
**Received low‐income subsidy**	122,363 (88.6)	123,658 (90.1)	40,929 (90.6)	0.017	0.012
**US region**				0.148	0.030
Northeast	21,614 (15.7)	21,348 (15.5)	6717 (14.9)		
Midwest	40,533 (29.4)	41,987 (30.6)	11,442 (25.3)		
South	61,122 (44.3)	59,614 (43.4)	22,039 (48.8)		
West	14,790 (10.7)	14,360 (10.5)	4994 (11.1)		
**Body mass index**				0.046	0.046
Underweight	7718 (5.6)	7630 (5.6)	2400 (5.3)		
Normal weight	41,364 (30.0)	39,538 (28.8)	13,234 (29.3)		
Overweight	39,843 (28.9)	39,894 (29.1)	12,883 (28.5)		
Obese	49,134 (35.6)	50,247 (36.6)	16,675 (36.9)		
**Clinical condition**					
Tobacco or alcohol use disorder	10,309 (7.5)	8844 (6.4)	4098 (9.1)	0.098	0.007
Mental health disorder	107,625 (78.0)	105,977 (77.2)	36,765 (81.4)	0.103	0.004
Diabetes	69,020 (50.0)	66,212 (48.2)	25,342 (56.1)	0.158	0.006
Cardiovascular disease	120,654 (87.4)	117,845 (85.8)	40,806 (90.3)	0.138	0.008
Hypertension	125,841 (91.2)	123,755 (90.1)	42,163 (93.3)	0.115	0.005
Pulmonary condition	104,067 (75.4)	99,330 (72.3)	36,639 (81.1)	0.208	0.010
Kidney disease	50,242 (36.4)	45,345 (33.0)	19,507 (43.2)	0.210	0.014
Liver disease	11,439 (8.3)	9536 (6.9)	4843 (10.7)	0.133	0.011
Gastrointestinal tract disorder	85,085 (61.6)	80,537 (58.7)	30,073 (66.5)	0.164	0.017
Injury	70,639 (51.2)	62,049 (45.2)	25,903 (57.3)	0.244	0.018
Neurodegenerative disorder	32,105 (23.3)	29,788 (21.7)	11,977 (26.5)	0.113	0.003
Drug use disorder	4751 (3.4)	4222 (3.1)	1750 (3.9)	0.044	0.008
BPSD	27,764 (20.1)	26,925 (19.6)	9561 (21.2)	0.038	0.002
Total number of comorbidities mean (SD)	30.3 (12.8)	28.0 (11.9)	34.2 (13.2)	0.493	0.019
**Health care use**					
Any hospital stay	52,606 (38.1)	39,793 (29.0)	23,091 (51.1)	0.465	0.033
Any ED visit	42,489 (30.8)	31,662 (23.1)	19,047 (42.1)	0.416	0.029
**Pain management**					
Drug or non‐drug pain intervention	133,170 (96.5)	134,662 (98.1)	42,101 (93.2)	0.242	0.001
Any procedure or therapy for managing chronic pain	74,695 (54.1)	69,338 (50.5)	26,958 (59.7)	0.185	0.011
Use of other pain medication					
Any adjuvant analgesic	69,390 (50.3)	70,507 (51.3)	24,302 (53.8)	0.049	0.003
Any prescription nonopioid	21,608 (15.7)	20,552 (15.0)	7554 (16.7)	0.048	0.002
Use of PRN pain medication	58,484 (42.4)	53,080 (38.7)	21,995 (48.7)	0.203	0.014
LTOT dosage, MME/d				0.294	0.041
< 20	99,873 (72.3)	92,274 (67.2)	36,079 (79.8)		
20–50	30,461 (22.1)	35,288 (25.7)	7717 (17.1)		
> 50	7725 (5.6)	9747 (7.1)	1396 (3.1)		
Use of long‐acting opioid	29,660 (21.5)	35,159 (25.6)	6236 (13.8)	0.300	0.015
**Medication use**					
Use of other CNS medication	109,305 (79.2)	107,997 (78.7)	36,349 (80.4)	0.044	0.004
Use of other ADRD medication	46,625 (33.8)	45,786 (33.3)	15,657 (34.6)	0.028	0.001
Polypharmacy	131,490 (95.2)	130,324 (94.9)	43,477 (96.2)	0.063	0.002
**Any opioid‐related side effect**	6989 (5.1)	7330 (5.3)	2505 (5.5)	0.009	0.003
**Dementia severity**				0.005	0.005
Mild	92,584 (67.1)	92,841 (67.6)	30,666 (67.9)		
Moderate	45,475 (32.9)	44,468 (32.4)	14,526 (32.1)		
**Baseline physical dependence**				0.093	0.000
No (ADL ≤ 9)	23,674 (17.1)	24,651 (18.0)	6624 (14.7)		
Mild (10 ≤ ADL ≤ 18)	27,137 (19.7)	26,550 (19.3)	8896 (19.7)		
Moderate (19 ≤ ADL ≤ 27)	59,392 (43.0)	57,855 (42.1)	20,290 (44.9)		
Severe (28 ≤ ADL)	27,856 (20.2)	28,253 (20.6)	9382 (20.8)		
**Baseline depression status**				0.114	0.000
No (PHQ‐9 ≤ 4)	109,553 (79.0)	109,628 (79.8)	36,141 (80.0)		
Mild (5 ≤ PHQ‐9 ≤ 9)	20,121(14.6)	19,796 (14.4)	6282 (13.9)		
Moderate (10 ≤ PHQ‐9 ≤ 14)	6446(4.7)	6047 (4.4)	2182 (4.8)		
Severe (15 ≤ PHQ‐9)	1939(1.4)	1838 (1.3)	587 (1.3)		
**Baseline pain status**				0.025	0.025
No	66,974(48.5)	67,918 (49.5)	21,904 (48.5)		
Mild	21,882(15.8)	21,790 (15.9)	7323 (16.2)		
Moderate	33,628(24.4)	32,271 (23.5)	11,199 (24.8)		
Severe	14,244(10.3)	13,998 (10.2)	4364 (9.7)		
**Time since ADRD diagnosis, years**				0.077	0.005
Mean (SD)	4.3 (3.5)	5.0 (3.7)	4.7 (3.7)		
**Year of index date**				0.103	0.040
2011	28,635(20.7)	22,496 (16.4)	6139 (13.6)		
2012	20,078(14.5)	16,681 (12.1)	5953 (13.2)		
2013	17,257 (12.5)	18,233 (13.3)	5944 (13.2)		
2014	16,284 (11.8)	16,857 (12.3)	6127 (13.6)		
2015	13,908 (10.1)	15,998 (11.7)	5015 (11.1)		
2016	13,721(9.9)	15,341 (11.2)	5128 (11.3)		
2017	13,036 (9.4)	14,348 (10.4)	4935 (10.9)		
2018	7984 (5.8)	9040 (6.6)	3110 (6.9)		
2019	7156 (5.2)	8315 (6.1)	2841 (6.3)		

Abbreviations: ADL, activities of daily living; ADRD, Alzheimer's disease and related dementias; BPSD, behavioral and psychological symptoms of dementia; CNS, central nervous system; ED, emergency department; IPTW, inverse probability of treatment weighting; LTOT, long‐term opioid therapy; MME, morphine milligram equivalent; PHQ‐9, patient health questionnaire‐9; PRN, as needed; SD, standard deviation; SDiff, standardized difference.

^a^ Clinical conditions and health care use were measured in the year and other characteristics were measured in the 6 months before index date (i.e., opioid discontinuation for discontinuers and frequency‐matched date for continuers).

^b^ Covariates with SDiff > 0.100 represent meaningful differences between case and control groups.

^c^ A patient could contribute to more than one episode.

^d^ Included Asian, Hispanic, Native American, and Pacific Islander.

Of 182,501 resident episodes with at least 1 year of follow‐up, 137,309 residents (75.2%) continued LTOT treatment, whereas 45,192 residents (24.7%) discontinued LTOT treatment. The baseline characteristics are given in Table 1. The discontinuers were more likely to have received diagnoses of clinical conditions, including diabetes (56.1% vs. 48.2%; SMD, 0.158), pulmonary condition (81.1% vs. 72.3%; SMD, 0.208), and injury (57.3% vs. 45.2%; SMD, 0.244) and to have experienced any hospitalization (51.1% vs. 29.0%; SMD, 0.465) and emergency department visit (42.1% vs. 23.1%; SMD, 0.416) during the 12‐month baseline period. Pain management during 6 months before the index date also differed between groups, with the discontinuers being less likely to receive any drug or non‐drug intervention for pain (93.2% vs. 98.1%; SMD, 0.242) but more likely to receive physical therapy (75.1% vs. 67.7%; SMD, 0.166), use PRN pain medications (48.7% vs. 38.7%; SMD, 0.203), and be prescribed an opioid dosage lower than 20 MME daily (79.8% vs. 67.2%, SMD, 0.294). After IPTW, the distributions of all measured baseline characteristics were well balanced between the continuers and discontinuers, with SMD for characteristics < 0.1.

### 3.1 Overall changes in clinical outcomes

Among residents with ADRD and chronic pain who received LTOT, the crude prevalence of individuals with worsening pain declined over time (32.5% to 27.4% from quarters 1 to 8; Table 2). After covariate adjustment, residents who discontinued versus continued LTOT had lower odds of worsening pain at the 1‐year (adjusted odds ratio [AOR], 0.71 [95% CI, 0.68–0.74]; *P* < 0.001) and 2‐year (AOR, 0.65 [95% CI, 0.61–0.68]; *P* < 0.001) follow‐up (Table 3). The interaction analysis indicated that discontinuation of LTOT was associated with a decreasing probability of worsening pain over time (AOR, 0.95 [95% CI, 0.94–0.96]; *P* for interaction < 0.001).

**TABLE 2:** Crude quarterly proportions of residents with worsening outcomes from baseline in the overall sample and by residents who discontinued or continued LTOT.

	Overall sample		Residents who discontinued LTOT		Residents who continued LTOT	
By quarter^a^	Number of resident episodes	% With worsening outcome from baseline	Number of resident episodes	% With worsening outcome from baseline	Number of resident episodes	% With worsening outcome from baseline
***Pain***						
Quarter 1	178,252	32.5	43,184	30.8	135,068	33.0
Quarter 2	179,958	31.3	44,526	28.3	135,432	32.3
Quarter 3	180,063	30.8	26,695	21.8	153,368	32.3
Quarter 4	180,395	29.8	28,948	20.7	151,447	31.5
Quarter 5	87,244	29.1	16,221	20.0	71,023	31.1
Quarter 6	87,206	28.4	17,991	20.2	69,215	30.6
Quarter 7	87,467	27.8	19,456	18.9	68,011	30.3
Quarter 8	87,471	27.4	20,856	18.0	66,615	30.3
***Physical function***						
Quarter 1	178,252	58.9	43,184	62.0	135,068	57.9
Quarter 2	179,958	59.9	44,526	61.1	135,432	59.5
Quarter 3	180,063	61.7	26,695	62.0	153,368	61.6
Quarter 4	180,395	63.9	28,948	64.2	151,447	63.9
Quarter 5	87,244	63.0	16,221	64.0	71,023	62.7
Quarter 6	87,206	64.6	17,991	65.5	69,215	64.4
Quarter 7	87,467	66.2	19,456	67.2	68,011	65.9
Quarter 8	87,471	68.0	20,856	68.7	66,615	67.9
***Depressive symptoms***	
Quarter 1	178,252	26.6	43,184	27.6	135,068	26.2
Quarter 2	179,958	28.9	44,526	28.3	135,432	29.1
Quarter 3	180,063	30.0	26,695	28.2	153,368	30.4
Quarter 4	180,395	31.2	28,948	29.7	151,447	31.5
Quarter 5	87,244	30.4	16,221	28.6	71,023	30.8
Quarter 6	87,206	30.7	17,991	28.8	69,215	31.2
Quarter 7	87,467	31.5	19,456	29.7	68,011	32.0
Quarter 8	87,471	31.0	20,856	29.7	66,615	32.7

Abbreviation: LTOT, long‐term opioid therapy.

^a^ Data for quarters 1 to 4 were derived from the eligible sample with at least 1 year of follow‐up; data for quarters 5 to 8, with at least 2 years of follow‐up.

**TABLE 3:** Quarterly associations of discontinuing LTOT with worsening clinical outcomes from baseline to follow‐up.

	Discontinuing versus continuing LTOT				Interaction of quarter time with LTOT discontinuation	
Outcome by quarters^a^	Crude OR (95% CI)	*P* value	Adjusted OR^b^ (95% CI)	*P* value	Adjusted OR^b^ (95% CI)	*P* value
***Worsening pain***						
Quarter 1	0.90 (0.88–0.93)	<0.001	0.90 (0.87–0.93)	<0.001	0.95 (0.94–0.96)	<0.001
Quarter 2	0.83 (0.81–0.85)	<0.001	0.86 (0.83–0.89)	<0.001		
Quarter 3	0.58 (0.56–0.60)	<0.001	0.73 (0.70–0.76)	<0.001		
Quarter 4	0.57 (0.55–0.59)	<0.001	0.71 (0.68–0.74)	<0.001		
Quarter 5	0.55 (0.53–0.58)	<0.001	0.71 (0.67–0.75)	<0.001		
Quarter 6	0.57 (0.55–0.60)	<0.001	0.74 (0.70–0.78)	<0.001		
Quarter 7	0.54 (0.52–0.56)	<0.001	0.69 (0.65–0.73)	<0.001		
Quarter 8	0.50 (0.48–0.52)	<0.001	0.65 (0.61–0.68)	<0.001		
***Worsening physical function***						
Quarter 1	1.18 (1.16–1.21)	<0.001	1.23 (1.20–1.26)	<0.001	0.94 (0.93‐0.96)	<0.001
Quarter 2	1.07 (1.04–1.09)	<0.001	1.12 (1.09–1.16)	<0.001		
Quarter 3	1.02 (0.99–1.04)	0.226	1.02 (0.98–1.06)	0.269		
Quarter 4	1.01 (0.99–1.04)	0.346	1.01 (0.98–1.05)	0.611		
Quarter 5	1.05 (1.02–1.09)	0.003	1.02 (0.97–1.07)	0.487		
Quarter 6	1.05 (1.01–1.09)	0.007	1.03 (0.98–1.08)	0.279		
Quarter 7	1.06 (1.03–1.10)	0.005	1.03 (0.98–1.08)	0.298		
Quarter 8	1.04 (1.00–1.07)	0.029	1.01 (0.96–1.06)	0.641		
***Worsening depressive symptoms***						
Quarter 1	1.07 (1.04–1.10)	<0.001	1.11 (1.08–1.15)	<0.001	0.96 (0.94–0.97)	<0.001
Quarter 2	0.96 (0.94–0.99)	0.001	1.00 (0.97–1.03)	0.930		
Quarter 3	0.90 (0.88–0.93)	<0.001	0.96 (0.93–1.00)	0.048		
Quarter 4	0.92 (0.89–0.94)	<0.001	0.95 (0.91–0.98)	0.006		
Quarter 5	0.90 (0.87–0.93)	<0.001	0.95 (0.90–1.00)	0.052		
Quarter 6	0.89 (0.86–0.92)	<0.001	0.93 (0.89–0.98)	0.006		
Quarter 7	0.90 (0.87 –0.93)	<0.001	0.95 (0.91–1.00)	0.051		
Quarter 8	0.87 (0.84–0.90)	<0.001	0.91 (0.87–0.95)	<0.001		

Abbreviations: CI, confidence interval; LTOT, long‐term opioid therapy; OR, odds ratio; PRN, as needed.

^a^ Data for quarters 1 to 4 were derived from the eligible sample with at least 1 year of follow‐up; data for quarters 5 to 8, with at least 2 years of follow‐up.

^b^ Adjusted baseline variables via inverse probability of treatment weighting and time‐varying confounders (including use of adjuvant analgesic, use of PRN pain medication, use of any pain intervention, use of central nervous system medication, and dementia severity) as covariates.

Among the study sample with ADRD, the crude prevalence values of residents with worsening physical function and depressive symptoms increased over time (58.9% to 68.0% and 26.6% to 31.0%, respectively, from quarters 1 to 8; Table 2). The proportions of residents with worsening physical function and depressive symptoms among LTOT discontinuers were initially higher but increased at a slower rate in later quarters relative to LTOT continuers (AOR, 0.94 [95% CI, 0.93–0.96] for physical function; AOR, 0.96 [95% CI, 0.94–0.97] for depressive symptoms; both *P* for interaction < 0.001; Tables 2 and 3). Patients who discontinued versus continued LTOT had 5% (AOR, 0.95 [95% CI, 0.91–0.98]; *P* < 0.001) lower odds of worsening depressive symptoms at the 1‐year follow‐up and 9% (AOR, 0.91 [95% CI, 0.87–0.95]; *P* < 0.001) lower odds at the 2‐year follow‐up but had no difference in worsening physical function at either the 1‐year (AOR, 1.01 [95% CI, 0.98–1.05]; *P* = 0.611) or 2‐year (AOR, 1.01 [95% CI, 0.96–1.06]; *P* = 0.641) follow‐up (Table 3).

### 3.2 Results of sensitivity and subgroup analyses

The sensitivity analysis using no opioid refills for at least 90 days to define opioid discontinuation yielded similar results to the main analysis for associations of worsening pain, physical function, and depressive symptoms in 1‐year and 2‐year follow‐up periods (Table S5 in supporting information). Consistent results were obtained for residents when stratified by baseline dementia severity (Table S6 in supporting information), types of pain conditions (Table S7 in supporting information), race and ethnicity (Table S8 in supporting information), and in most of the subgroups stratified according to no‐to‐mild or moderate‐to‐severe pain, physical function, and depressive symptoms at baseline (Tables S9–S11 in supporting information). Specifically, in the subgroup of residents with no‐to‐mild pain at baseline, the odds of experiencing worsening pain among the LTOT discontinuers versus continuers was higher in quarter 1 but were lower thereafter throughout follow‐up periods.

## 4 DISCUSSION

In this cohort study assessing national longitudinal data of older Medicare NH residents with mild or moderate ADRD and chronic non‐cancer pain who received LTOT, residents who discontinued versus continued LTOT had lower odds of experiencing worsening pain and depressive symptoms with no difference in physical function at the 1‐year and 2‐year follow‐up periods. Findings in both sensitivity and subgroup analyses were consistent to those of the main analyses. Our results suggested that discontinuation of LTOT was associated with lower odds of worsening pain and depressive symptoms, with no difference in physical function in both the short and long terms.

Limited longitudinal population‐based studies have been conducted to examine the associations of LTOT discontinuation with clinical outcomes of pain, physical function, and depression status among older adults, particularly those with ADRD.^13^ The existing literature has primarily focused on young or mixed young and older populations and has studied the association of LTOT discontinuation with opioid overdose, which is rare among patients with ADRD.^31^, ^32^ Other prior studies have examined clinical consequences after tapering high‐dose opioids (e.g., ≥ 50 MME daily) among LTOT recipients,^32^, ^33^, ^34^ a clinical practice that is infrequent among patients with ADRD given that the majority of affected patients are prescribed a low opioid dosage (< 20 MME daily).

The present study provides evidence of a possible latency period for changes in physical function and depressive symptoms among patients with ADRD after discontinuation of LTOT. We observed positive associations between discontinuation of LTOT and worse physical function and depressive outcomes in the first or second quarters but protected or non‐significant associations in later quarters during follow‐up years. While the underlying mechanisms for these observations are not entirely clear, there are at least two plausible explanations. First, patients who discontinue LTOT may immediately experience opioid withdrawal symptoms, resulting in worsening of physical and depressive function in initial follow‐up quarters. Opioid withdrawal symptoms, however, are expected to be time limited, and as the symptoms subside, the chance of worsening physical and depressive function may decrease over time. Second, the positive associations with worsening physical and depressive function in the initial quarters may be because many residents had already experienced unstable, deteriorated physical and mental health conditions at baseline before their LTOT was discontinued. This explanation is supported by our baseline data that LTOT discontinuers versus continuers were in general sicker and more likely to have physical and mental comorbidities and experience hospitalization and ED visits. The preclinical unstable health conditions may have resulted in the discontinuation of LTOT and in subsequent worse physical function and depressive symptoms during early follow‐up, a phenomenon known as reverse causality. The differences in the direction of the associations between early and late quarterly follow‐ups also suggested that the latency for changes in physical function and depressive symptoms may range between 3 and 6 months after discontinuation of LTOT among residents with ADRD.

The present study provides referential data for clinicians weighing the clinical benefits of continuing versus discontinuing LTOT for residents with mild or moderate ADRD and chronic pain. For example, worsening pain decreased over time in both LTOT continuers and discontinuers, but the reduction appeared to be greater among residents who discontinued LTOT. Regardless of continuing or discontinuing LTOT, physical function and depressive symptoms worsened over time among these NH residents with ADRD. Nevertheless, residents who discontinued (vs. continued) LTOT had a significantly slower progression to worsening depressive symptoms in the short and long terms, supported by clinical observations that long‐term opioid use could lead to depression.^35^ We observed no group difference in short‐ and long‐term physical function, suggesting that discontinuation of LTOT may have no association with physical function among residents with ADRD. Further studies are needed to examine safety outcomes (e.g., hospitalization or falls) of discontinuation versus continuation of LTOT to provide comprehensive benefit–risk evidence. Overall, tailoring pain treatment and management to individual clinical need is essential to achieve treatment goals aligned with patients with ADRD and chronic pain.

## 5 LIMITATIONS

First, Medicare claims data lack information on medical notes on reasons (e.g., drug contraindications, lack of opioid efficacy, or improved pain control) that may justify the discontinuation of LTOT. These unmeasured clinical reasons could potentially bias the results. In our study, LTOT discontinuers (vs. continuers) might have been on a better pain trajectory, triggering the decision to discontinue LTOT and leading to lower odds of worsening pain during follow‐up periods. Additional approaches (e.g., instrumental variable methods) that account for unmeasured confounders and data that include clinical justifications for discontinuation of LTOT are needed to confirm our findings. Second, information is unavailable about whether discontinuation was initiated by residents or their caregivers and the clinical appropriateness of LTOT discontinuation. Third, because we relied on resident‐reported clinical outcomes, self‐report bias is possible; however, such bias is likely non‐differential between LTOT continuers and discontinuers. Fourth, Medicare prescription data do not capture self‐paid prescriptions or opioids covered by non‐Medicare programs. Finally, our results can be generalized only to older NH residents with mild‐to‐moderate ADRD who survive for 2 years or longer and are able to communicate.

## 6 CONCLUSIONS

Among older NH residents with mild‐to‐moderate ADRD and receipt of LTOT, those who discontinued (vs. continued) LTOT were less likely to experience worsening pain and depressive symptoms and had no difference in physical function at 1‐ and 2‐year follow‐ups. Further studies examining safety outcomes associated with discontinuation versus continuation of LTOT among patients with ADRD are warranted.

## CONFLICT OF INTEREST STATEMENT

All authors have no conflicts of interest and nothing to disclose. Author disclosures are available in the supporting information.

## CONSENT STATEMENT

The Ohio State University's Institutional Review Board approved and waived patient informed consent and HIPAA authorization for this study because of minimal risk and lack of feasibility to contact Medicare subjects.

## Supporting information

Supporting Information

Supporting Information
