# Supplementary material for: Pain intensity, physical function, and depressive symptoms associated with discontinuing long‐term opioid therapy in older adults with Alzheimer's disease and related dementias
Source: Alzheimers Dement. 2023 Oct 19;20(2):1026–37. doi: 10.1002/alz.13489 (PMC10916940; doi:10.1002/alz.13489)
Supplement: Supplementary file 1 — Supporting Information [file ALZ-20-1026-s001.docx]

**Supplementary Online Content**

Wei Y-JJ, Winterstein AG, Schmidt S, et al. Pain intensity, physical function, and depressive symptoms associated with discontinuing long-term opioid therapy in older adults with Alzheimer disease and related dementias.

**Table A.1.** Medications of Interest Considered in the Study

**Table A.2**. *ICD-9-CM, ICD-10-CM,* or Procedure Codes for Disease Conditions and Service Care Considered in the Study

**Table A.3**. Study Covariates, Definitions, and Measurement Sources and Windows

**Table A.4.** Clinical and Demographic Characteristics of Eligible Patients With at Least 2 Years of Follow-up, Overall and by Continuation vs Discontinuation of Long-term Opioid Therapy

**Table A.5.** Quarterly Associations of Discontinuing Long-term Opioid Therapy, Defined as a Gap of ≥90 Consecutive Days, With Worsening Clinical Outcomes from Baseline to Follow-Up

**Table A.6.** Quarterly Associations of Discontinuing Long-term Opioid Therapy With Worsening Clinical Outcomes, Stratified by Residents With Mild or Moderate ADRD at Baseline

**Table A.7.** Quarterly Associations of Discontinuing Long-term Opioid Therapy With Worsening Clinical Outcomes, Stratified by Residents With Types of Pain Conditions at Baseline

**Table A.8.** Quarterly Associations of Discontinuing Long-term Opioid Therapy With Worsening Clinical Outcomes, Stratified by Race and Ethnicity

**Table A.9.** Quarterly Associations of Discontinuing Long-term Opioid Therapy With Worsening Clinical Outcomes, Stratified According to No-to-Mild or Moderate-to-Severe Pain at Baseline

**Table A.10.** Quarterly Associations of Discontinuing Long-term Opioid Therapy With Worsening Clinical Outcomes, Stratified According to No-to-Mild or Moderate-to-Severe Physical Function at Baseline

**Table A.11.** Quarterly Associations of Discontinuing Long-term Opioid Therapy With Worsening Clinical Outcomes, Stratified According to No-to-Mild or Moderate-to-Severe Depressive Symptoms at Baseline

**Figure A.1.** Schematic Diagram of Study Design, Including Time Period for Identifying Cohort Entry, Index Date for Exposure Status, Eligibility Criteria, and 1-Year Outcome Follow-up

**Table A.1.** Medications of Interest Considered in the Study

| **Medication Class** | **Individual Medication** |
| --- | --- |
| **Opioids** | butorphanol, codeine, dihydrocodeine, fentanyl, hydrocodone, hydromorphone, levorphanol, meperidine, methadone, morphine, nalbuphine, opium, oxycodone, oxymorphone, pentazocine, remifentanil, sufentanil, tramadol.  *Excluding injectable opioids used primarily for inpatients, rectal dosage forms that are rarely used, opioids used as cold medicines (eg, promethazine/codeine syrup) to suppress cough, and buprenorphine in sublingual form and combined buprenorphine-naloxone products, which are used for addiction treatment. |
| **Nonopioids** |  |
| Analgesics and antipyretics | acetaminophen, salicylamide, sodium thiosalicylate, ziconotide |
| NSAIDs | celecoxib, diclofenac potassium/sodium, etodolac, fenoprofen, flurbiprofen, ibuprofen, indomethacin, ketoprofen, ketorolac, meclofenamate, mefenamic acid, meloxicam, nabumetone, naproxen, oxaprozin, piroxicam, sulindac, tolmetin |
| Salicylates | Aspirin, diflunisal, choline magnesium trisalicylate, magnesium salicylate, and salsalate |
| **Adjuvant analgesics** |  |
| SNRI antidepressants | Duloxetine, venlafaxine, desvenlafaxine, Milnacipran, Levomilnacipran |
| Tricyclic antidepressants | amitriptyline, clomipramine, imipramine, doxepin, trimipramine, amoxapine, maprotiline, nortriptyline, desipramine, protriptyline, |
| Anticonvulsants (for pain treatment) | carbamazepine, fosphenytoin, gabapentin, lacosamide, lamotrigine, levetiracetam, oxcarbazepine, phenytoin, pregabalin, topiramate, and valproate |
| Skeletal muscle relaxants | baclofen, carisoprodol, chlorzoxazone, cyclobenzaprine, dantrolene, diazepam, metaxalone, methocarbamol, orphenadrine, tizanidine |
| **Other CNS Medications** |  |
| Benzodiazepines | alprazolam, estazolam, lorazepam, oxazepam, temazepam, triazolam, midazolam, chlordiazepoxide, clobazam, clonazepam, clorazepate, diazepam, prazepam, flurazepam, quazepam. |
| Antipsychotics | acetophenazine, chlorpromazine, droperidol, fluphenazine, haloperidol, loxapine, mesoridazine, molindone, perphenazine, pimozide, prochlorperazine, thioridazine, thiothixene, trifluoperazine, aripiprazole, asenapine, brexpiprazole, cariprazine, clozapine, iloperidone, fanapt, lurasidone, olanzapine, paliperidone, primavaserin, quetiapine, risperidone, ziprasidone. |
| Sedative-hypnotics | buspirone, meprobamate, eszopiclone, zaleplon, zolpidem, suvorexant, hydroxyzine, diphenhydramine, ramelteon, tasimelteon, amobarbital, butabarbital, pentobarbital, secobarbital, phenobarbital, mephobarbital, chloral hydrate, lemborexant |
| Antidepressants (excluding drugs for pain treatment) | mirtazapine, bupropion, isocarboxazid, phenelzine, tranylcypromine, selegiline, rasagiline, nefazodone, trazodone, citalopram, escitalopram, fluoxetine, fluvoxamine, paroxetine, sertraline, vilazodone, vortioxetine, maprotiline |
| Anticonvulsants (excluding those for pain treatment) | [acetazolamide](https://www.medicines.org.uk/emc/search?q=Acetazolamide), brivaracetam, cenobamate, eslicarbazepine acetate, ethadione, ethotoin, ethosuximide, ezogabine, felbamate, fenfluramine, mephenytoin, methohexital, methsuximide, magnesium sulfate, perampanel, paramethadione, phenobarbital, primidone, rufinamide, stiripentol, sultiame, tiagabine, trimethadione, vigabatrin, zonisamide |
| **Antidementia drugs** | donepezil, galantamine, memantine, rivastigmine, tacrine (discontinued in 2013) |

Abbreviations: NSAID, nonsteroidal anti-inflammatory; SNRI, serotonin and norepinephrine reuptake inhibitor; CNS, central nervous system.

**Table A.2**. *ICD-9-CM, ICD-10-CM,* or Procedure Codes for Disease Conditions and Service Care Considered in the Study

| **Disease, Condition, or Service Care** | ***ICD-9-CM, ICD-10-CM,* or Procedure Code** | **Algorithm** |
| --- | --- | --- |
| ADRD | 331.0, 331.11, 331.19, 331.2, 331.7, 290.0, 290.10, 290.11, 290.12, 290.13, 290.20, 290.21, 290.3, 290.40, 290.41, 290.42, 290.43, 294.0, 294.10, 294.11, 294.20, 294.21, 294.8, 797, F01.50, F01.51, F02.80, F02.81, F03.90, F03.91, F04, F05, F06.1, F06.8, G13.8, G30.0, G30.1, G30.8, G30.9, G31.01, G31.09, G31.1, G31.2, G94, R41.81, R54 | At least 1 inpatient, SNF, HHA, HOP, or Carrier claim with disease code in any diagnostic position during a 3-year period. |
| Chronic pain |  |  |
| Musculoskeletal | 274.x, 710.x-729.x (exclude 723.4, 724.3, 724.4, 729.1, 729.2),  A18.01-A18.02, A52.16, D48.1, E08.61x, E09.61x, E10.61x, E11.61x, E13.61x, M00-M02, M04.02-M04.09, M05-M19, M1A, M20.10, M21.61-M21.62, M22-M25, M32-M36, M43.2-M43.8X9, M45-M48, M49.80, M50, M51, M53, M54, M60.0-M60.2, M61-M63, M65-M67, M70-M72, M75-M77, M79, M96.1, M99.2-M99.7, N20.0, Q68.6, R25.2, R26.2, R29.8x | At least 1 inpatient, SNF, HHA, HOP, or carrier claim with disease code in any diagnostic position |
| Neuropathic | 053.1x, 249.6, 250.6, 307.89, 336.x, 337.x, 338.0, 340, 350.x, 351.x, 352.1, 353.x-355.x, 357.1, 357.2-357.4, 357.8, 357.9, 723.4, 724.3, 724.4, 729.1, 729.2,  A52.15, B02 (exclude B02.1), EXX.4, EXX.610, EXX.65 (where X in “08”-“13”), E10.4, F45.42, G13.0, G13.1, G32.0, G35, G50- G52.1, G54-G59, G61.8, G61.9, G62.8, G62.9, G63-G65, G89.0, G90.0, G90.5, G95, G99.0-G99.2, M05.5, M54.13-M54.18, M54.3, M54.4, M60.8, M60.9, M79.1, M79.2, M79.7 |  |
| Idiopathic | 338.2, 338.4, 780.96, G89, R52 |  |
| Cancer diagnosis | CCS11-CCS43 | HCUP CCS for *ICD-9-CM or ICD-10-CM* |
| Hospice care | Admission date of hospice claims | At least 1 hospice claim in any diagnostic position |
| Palliative Care | DX: V 66.7  Provide specialty code: 17 | At least 1 inpatient, SNF, HHA, HOP, carrier, or DME claim with disease code in any diagnostic position; or at least 1 inpatient, SNF, HHA, HOP with provider specialty code |
| Tobacco use disorder | 305.1, 649.0x, 989.84, F17.x, O99.33x, T65.21xA, Z72.0 | At least 1 inpatient, SNF, HHA, HOP, carrier, or DME claim with disease code in any diagnostic position |
| Alcohol use disorder | 291.x, 303.x, 305.0x, 357.5, 425.5, 535.3x, 571.0-571.3, 760.71, 980.0, V65.42, V79.1, E860.0, F10.x (excluding F10.11, F10.13, F10.21, F10.93), G62.1, I42.6, K29.2x, K70.x, P04.3, Q86.0, T51.0XxA, Z71.4x |  |
| **Clinical condition** |  |  |
| Mental disorder | Anxiety disorders (CCS 651), mood disorders (CCS 657), schizophrenia and other psychotic disorders (CCS 659) | HCUP CCS for *ICD-9-CM or ICD-10-CM* |
| Diabetes | Diabetes mellitus without complication (CCS 49), diabetes mellitus with complications (CCS 50) |  |
| Cardiovascular disease | Heart valve disorders (CCS 96), coronary atherosclerosis and other heart disease (CCS 101), pulmonary heart disease (CCS 103), cardiac dysrhythmias (CCS 106), congestive heart failure; nonhypertensive (CCS 108), acute cerebrovascular disease (CCS 109), occlusion or stenosis of precerebral arteries (CCS 110), other and ill-defined cerebrovascular disease (CCS 111), peripheral and visceral atherosclerosis (CCS 114) |  |
| Hypertension | essential hypertension (CCS 98), hypertension with complications, and secondary hypertension (CCS 99) |  |
| Pulmonary condition | Pneumonia (except that caused by tuberculosis or sexually transmitted disease) (CCS 122), acute bronchitis (CCS 125), other upper respiratory infections (CCS 126), chronic obstructive pulmonary disease and bronchiectasis (CCS 127), asthma (CCS 128), pleurisy; pneumothorax; pulmonary collapse (CCS 130), respiratory failure; insufficiency; arrest (CCS 131), other lower respiratory disease (CCS 133) |  |
| Kidney disease | Nephritis; nephritis; renal sclerosis (CCS 156), acute and unspecified renal failure (CCS 157), chronic kidney disease (CCS 158), other diseases of kidney and ureters (CCS 161) |  |
| Gastrointestinal tract disorder | Gastrointestinal hemorrhage (CCS 153), other gastrointestinal disorders (CCS 155), digestive congenital anomalies (CCS 214) |  |
| Liver disease | Liver diseases (CCS 151) |  |
| Injury | Pathological fracture (CCS 207), fracture of neck of femur (hip) (CCS 226), skull and face fractures (CCS 228), fracture of upper limb (CCS 229), fracture of lower limb (CCS 230), other fractures (CCS 231), e-codes: fall (CCS 2603). |  |
| Neurodegenerative disorder | Parkinson disease (CCS 79), other hereditary and degenerative nervous system conditions (CCS 81) |  |
| Drug use disorder | Chronic Condition Data Warehouse, Drug Use Disorder Algorithm^1^ | At least 1 inpatient, SNF, HHA, HOP, carrier, or DME claim with disease code |
| BPSD | Presence or absence of any of the following symptoms: psychotic (delusions or hallucinations) and disruptive behaviors (rejection of care, or physical, verbal, or other aggressive behaviors) | MDS 3.0 |
| *Procedures* |  |  |
| Medical procedures and therapies for chronic pain management | 20550-20552, 20560, 20561, 28899, 20999, 62281, 62324, 62325, 64405, 64408, 64415, 64417, 64418, 64420, 64421, 64425, 64430, 64435, 64445-64451, 64461-64463, 64505, 64510, 64517, 64520, 64530, 64620, 64632, 64660, 64999, 64450, 76881, 76882, 76942, 76999, 90901, 90912, 90913, 92506, 97001-97004, 97010, 97012, 97016, 97018, 97022, 97024, 97026, 97028, 97032-97036, 97039, 97110, 97112, 97113, 97116, 97124, 97139, 97140, 97150, 97530, 97542, 97597, 97598, 97760, 97799, 97610, 97161-97168, 97810, 97811, 97813, 97814, G0281, G0282, G0283, G0329, 0019T, 98940, 98941, 98942, 98943 | At least 1 inpatient, SNF, HHA, HOP, carrier, or DME claim with disease code |

Abbreviations: ADRD, Alzheimer disease and related dementias; BPSD, behavioral and psychological symptoms of dementia; CCS, Clinical Classification Software; DME, Duration Medical Equipment; HCUP, Healthcare Cost and Utilization Project; HHA, home health agency; HOP, hospital outpatient; *ICD-9-CM and ICD-10-CM*, *International Classification of Diseases, Ninth or Tenth Revision, Clinical Modification*; SNF, skilled nursing facility.

^1^https://www2.ccwdata.org/web/guest/condition-categories-other

**Table A.3**. Study Covariates, Definitions, and Measurement Sources and Windows

| **Covariate** | **Measurement** | **Measurement window** | **Data source** |
| --- | --- | --- | --- |
| **Demographic characteristic** |  |  |  |
| Age | Classified into 3 age groups: 65-74, 75-84, and ≥85 years | On index date | Medicare Beneficiary Summary File |
| Sex | Male or female | On index date | Medicare Beneficiary Summary File |
| Race/ethnicity | Measured based on the Medicare-Research Triangle Institute race code and grouped into 3 groups: White, Black, and other (including Hispanic, Asian, Pacific Islander, and Native American individuals) | On index date | Medicare Beneficiary Summary File |
| US geographic region | Northeast, Midwest, West, and South | On index date | Medicare Beneficiary Summary File |
| Receipt of low-income subsidy (LIS) | LIS receipt was defined as having at least 6 months of receiving LIS; otherwise, non-LIS receipt | 12 months before index date | Medicare Beneficiary Summary File |
| Body mass index | MDS-documented weight in kilograms divided by the MDS-documented height in meters squared and categorized as underweight [BMI <18.5], normal [BMI 18.5-24.9], obese [BMI 25.0-29.9], and overweight [BMI ≥30] | 6 months before index date | MDS 3.0 |
| Years of living with ADRD | Years of living with ADRD since the first diagnosis in Medicare data | Prior to index date | Medicare Chronic Condition file |
| **Clinical condition** |  |  |  |
| Tobacco or alcohol use disorder | Presence or absence of diagnosis | 12 months before index date | Medicare Parts A and B |
| Drug use disorder | Presence or absence of diagnosis | 12 months before index date | Medicare Parts A and B |
| Conditions affecting opioid treatment |  |  |  |
| Hypertension | Presence or absence of diagnosis | 12 months before index date | Medicare Parts A and B |
| Diabetes | Presence or absence of diagnosis | 12 months before index date | Medicare Parts A and B |
| Cardiovascular disease | Presence or absence of diagnosis | 12 months before index date | Medicare Parts A and B |
| Pulmonary condition | Presence or absence of diagnosis | 12 months before index date | Medicare Parts A and B |
| Mental health disorder | Presence or absence of diagnosis | 12 months before index date | Medicare Parts A and B |
| Gastrointestinal disease | Presence or absence of diagnosis | 12 months before index date | Medicare Parts A and B |
| Injury | Presence or absence of diagnosis | 12 months before index date | Medicare Parts A and B |
| Kidney disease | Presence or absence of diagnosis | 12 months before index date | Medicare Parts A and B |
| Liver disease | Presence or absence of diagnosis | 12 months before index date | Medicare Parts A and B |
| Neurodegenerative diseases | Presence or absence of diagnosis | 12 months before index date | Medicare Parts A and B |
| Behavioral and psychological symptoms | Presence or absence of the symptoms | 12 months before index date | MDS 3.0 |
| Total number of comorbidities | Sum of the number of AHRQ CCS categories excluding aforementioned pain and clinical conditions | 12 months before index date | Medicare Parts A and B |
| **Pain management** |  |  |  |
| Use of any pain management | Yes vs no | 6 months before index date | MDS 3.0 |
| Use of PRN pain medications | Yes vs no | 6 months before index date | MDS 3.0 |
| Receipt of procedures or therapies for chronic pain management | Presence or absence of CPT codes | 6 months before index date | Medicare Parts A and B |
| Opioid dosage | Classified as < 20, 20-50, > 50 MME/day | 6 months before index date | Medicare Part D |
| Use of long-acting opioids | Yes vs no (list of long-acting) | 6 months before index date | Medicare Part D |
| Use of prescription nonopioids | Yes vs no | 6 months before index date | Medicare Part D |
| Use of adjuvant analgesics | Yes vs no | 6 months before index date | Medicare Part D |
| **Medication utilization** |  |  |  |
| Use of other CNS medication | Yes vs no | 6 months before index date | Medicare Part D |
| Use of antidementia drug | Yes vs no | 6 months before index date | Medicare Part D |
| Polypharmacy | Yes vs no | 6 months before index date | Medicare Part D |
| **Dementia severity** | Mild (BIMS: 8-12 or CPS: 0-2) or moderate (BIMS: 0-7 or CPS: 3-4) dementia | 6 months before index date | MDS 3.0 |
| **Opioid adverse effects** | Yes vs no | 6 months before index date | MDS 3.0 |
| **Baseline physical function** | No (ADL score ≤ 9), mild (10 ≤ ADL ≤ 18), moderate (19 ≤ ADL ≤ 27), or severe (ADL ≥ 28) | 6 months before index date | MDS 3.0 |
| **Baseline depressive symptoms** | No (PHQ-9 score ≤ 4), mild (5 ≤ PHQ-9 ≤ 9), moderate (10 ≤ PHQ-9 ≤ 14), or severe (PHQ-9 ≥ 15) | 6 months before index date | MDS 3.0 |
| **Baseline pain** | No, mild, moderate, or severe | 6 months before index date | MDS 3.0 |
| **Year of index date** | Range from 1 (2011) to 9 (2019) for the cohort with ≥1 year of follow-up; range from 1 (2011) to 8 (2018) for the sub-cohort with ≥2 years of follow-up; | On index date | Medicare Part D |

Abbreviations: ADL, activities of daily living; ADRD, Alzheimer disease and related dementias; AHRQ CCS, Agency for Healthcare Research and Quality Clinical Classifications Software; BIMS, Brief Interview for Mental Status; BMI, body mass index; CNS, central nervous system; CPS, Cognitive Performance Scale; CPT, Current Procedural Terminology; MME, morphine milligram equivalents; PHQ-9, Patient Health Questionnaire-9; PRN, as needed; MDS, Minimum Data Set.

**Table A.4.** Clinical and Demographic Characteristics of Eligible Patients With at Least 2 Years of Follow-up, Overall and by Continuation vs Discontinuation of Long-term Opioid Therapy

|  | No. (%)^a^ |  |  | SDiff ^b^ |  |
| --- | --- | --- | --- | --- | --- |
| **Characteristic** | Overall sample  (n=78127) | Continuers  (n=65973)^c^ | Discontinuers  (n=22416)^c^ | Before  IPTW | After  IPTW |
| **Age, y** |  |  |  |  |  |
| Mean (SD) | 82.4 (8.6) | 82.7 (8.6) | 81.8 (8.6) | 0.091 | 0.000 |
| 65-74 | 16778 (21.5) | 13507 (20.5) | 5261(23.5) |  |  |
| 75-84 | 26516 (33.9) | 22466 (34.1) | 7898(35.2) |  |  |
| ≥85 | 34833 (44.6) | 30000 (45.5) | 9257(41.3) |  |  |
| **Female** | 64055 (82.0) | 54846 (83.1) | 17790(79.4) | 0.097 | 0.08 |
| **Male** | 14072 (18.0) | 11127 (16.9) | 4626 (20.6) |  |  |
| **Race and ethnicity** |  |  |  | 0.149 | 0.041 |
| White | 63773 (81.6) | 54775 (83.0) | 17280(77.1) |  |  |
| Black | 9380 (12.0) | 7440 (11.3) | 3305(14.7) |  |  |
| Other^d^ | 4974 (6.4)) | 3758 (5.7) | 1831(8.2) |  |  |
| **Receiving low-income subsidy** | 69979 (89.6) | 59502 (90.2) | 20360 (90.8) | 0.022 | 0.013 |
| **US Region** |  |  |  | 0.126 | 0.032 |
| Northeast | 12129 (15.5) | 10252 (15.5) | 3337 (14.9) |  |  |
| Midwest | 22436 (28.7) | 19794 (30.0) | 5573 (24.9) |  |  |
| South | 35159 (45.0) | 28967 (43.9) | 10989(49.0) |  |  |
| West | 8403 (10.8) | 6960 (10.5) | 2517 (11.2) |  |  |
| **Body Mass Index** |  |  |  | 0.025 | 0.025 |
| Underweight | 3849 (4.9) | 3252 (4.9) | 1088 (4.9) |  |  |
| Normal weight | 22422 (28.7) | 18391 (27.9) | 6530 (29.1) |  |  |
| Overweight | 23040 (29.5) | 19459 (29.5) | 6521(29.1) |  |  |
| Obese | 28816 (36.9) | 24871 (37.7) | 8277 (36.9) |  |  |
| **Clinical condition** |  |  |  |  |  |
| Tobacco or alcohol use disorder | 5667 (7.3) | 4228 (6.4) | 2034 (9.1) | 0.099 | 0.008 |
| Mental health disorder | 60694 (77.7) | 50720 (76.9) | 18150 (81.0) | 0.100 | 0.001 |
| Diabetes | 38972 (49.9) | 31740 (48.1) | 12443 (55.5) | 0.149 | 0.002 |
| Cardiovascular disease | 67443 (86.3) | 56049 (85.0) | 20063(89.5) | 0.137 | 0.007 |
| Hypertension | 70824 (90.7) | 59282 (89.9) | 20782 (92.7) | 0.101 | 0.003 |
| Pulmonary condition | 57941 (74.2) | 47128 (71.4) | 17965 (80.1) | 0.204 | 0.007 |
| Kidney disease | 26625 (34.1) | 20807 (31.5) | 9016 (40.2) | 0.182 | 0.013 |
| Liver disease | 6215 (8.0) | 4584 (6.9) | 2275 (10.1) | 0.115 | 0.007 |
| Gastrointestinal tract disorder | 46782 (59.9) | 37973 (57.6) | 14630 (65.3) | 0.159 | 0.019 |
| Injury | 38930 (49.8) | 30011 (45.5) | 12769 (57.0) | 0.231 | 0.017 |
| Neurodegenerative disorder | 17560 (22.5) | 14063 (21.3) | 5713 (25.5) | 0.099 | 0.005 |
| Drug use disorder | 2529 (3.2) | 1968 (3.0) | 807 (3.6) | 0.035 | 0.009 |
| BPSD | 14519 (18.6) | 12790 (19.4) | 4663 (20.8) | 0.035 | 0.003 |
| Total number of comorbidities [Mean (SD)] | 29.6 (12.4) | 27.8 (11.8) | 33.4 (12.9) | 0.455 | 0.031 |
| **Health care use** |  |  |  |  |  |
| Any hospital stay | 28899 (37.0) | 19735 (29.9) | 11413 (50.9) | 0.438 | 0.028 |
| Any ED visit | 23191 (29.7) | 15691 (23.8) | 9297 (41.5) | 0.384 | 0.029 |
| **Pain management** |  |  |  |  |  |
| Drug or non-drug pain intervention | 75323 (96.4) | 64614 (97.9) | 20780 (92.7) | 0.250 | 0.006 |
| Any procedure or therapy for managing chronic pain | 41210 (52.8) | 33002 (50.6) | 13177 (58.8) | 0.177 | 0.012 |
| Use of other pain medication |  |  |  |  |  |
| Any adjuvant analgesic | 39127 (50.1) | 33385 (50.6) | 11785 (52.6) | 0.039 | 0.010 |
| Any prescription nonopioid | 12834 (16.4) | 10447 (15.8) | 3972 (17.7) | 0.050 | 0.006 |
| Use of PRN pain medication | 33054 (42.3) | 25815 (39.1) | 10995 (49.0) | 0.201 | 0.015 |
| Dosage of long-term opioid therapy |  |  |  | 0.276 | 0.045 |
| <20 MME daily | 56381 (72.2) | 44790 (67.9) | 18020 (80.4) |  |  |
| 20-50 MME daily | 17410 (22.3) | 16652 (25.2) | 3763 (16.8) |  |  |
| >50 MME daily | 4336 (5.5) | 4531 (6.9) | 633 (2.8) |  |  |
| Use of long-acting opioid | 16656 (21.3) | 16397 (24.9) | 3048 (13.6) | 0.289 | 0.009 |
| **Medication use** |  |  |  |  |  |
| Use of other CNS medication | 62088 (79.5) | 52179 (79.1) | 18098 (80.7) | 0.041 | 0.001 |
| Use of other ADRD medication | 27196 (22.3) | 22706 (34.4) | 7977 (35.6) | 0.025 | 0.005 |
| Polypharmacy | 74345 (95.2) | 62597 (94.9) | 21522 (96.0) | 0.054 | 0.000 |
| **Any opioid-related side effect** | 3266 (4.2) | 2828 (4.3) | 1030 (4.6) | 0.015 | 0.001 |
| **Dementia severity** |  |  |  | 0.007 | 0.006 |
| Mild | 53414 (68.4) | 45413 (68.8) | 15355 (68.5) |  |  |
| Moderate | 24713 (31.6) | 20560 (31.2) | 7061 (31.5) |  |  |
| **Baseline physical dependence** |  |  |  | 0.108 | 0.000 |
| No (ADL score ≤9) | 14907 (19.1) | 13230 (20.1) | 3644 (16.3) |  |  |
| Mild (10 ≤ ADL ≤ 18) | 16011 (20.5) | 13366 (20.3) | 4626 (20.6) |  |  |
| Moderate (19 ≤ ADL ≤ 27) | 32563 (41.7) | 26964 (40.9) | 9811 (43.8) |  |  |
| Severe (ADL ≥28) | 14646 (18.7) | 12413 (18.8) | 4335 (19.3) |  |  |
| **Baseline depression status** |  |  |  | 0.114 | 0.000 |
| No (PHQ-9 score ≤4) | 62207 (79.6) | 52697 (79.9) | 17968 (80.2) |  |  |
| Mild (5 ≤ PHQ-9 ≤ 9) | 11285 (14.4) | 9448 (14.3) | 3125 (13.9) |  |  |
| Moderate (10 ≤ PHQ-9 ≤ 14) | 3573 (4.6) | 2914 (4.4) | 1058 (4.7) |  |  |
| Severe (PHQ-9 ≥15) | 1062(1.4) | 914 (1.4) | 265 (1.2) |  |  |
| **Baseline pain status** |  |  |  | 0.038 | 0.025 |
| No | 37535 (48.0) | 32032 (48.6) | 10786 (48.1) |  |  |
| Mild | 12218 (15.6) | 10308 (15.6) | 3573 (15.9) |  |  |
| Moderate | 19276 (24.7) | 15899 (24.1) | 5647 (25.2) |  |  |
| Severe | 8355 (10.7) | 7106 (10.8) | 4364 (19.5) |  |  |
| **Time since ADRD diagnosis, y** |  |  |  |  |  |
| Mean (SD) | 4.4 (3.5) | 4.8 (3.6) | 4.6 (3.6) | 0.078 | 0.014 |
| **Year of index date** |  |  |  | 0.103 | 0.000 |
| 2011 | 18971 (24.3) | 14937 (22.6) | 4034 (18.0) |  |  |
| 2012 | 12477 (16.0) | 9037 (13.7) | 3440 (15.3) |  |  |
| 2013 | 9660 (12.4) | 7737 (11.7) | 2947 (13.1) |  |  |
| 2014 | 10099 (12.9) | 9352 (14.2) | 3369 (15.0) |  |  |
| 2015 | 8406 (10.8) | 7866 (11.9) | 2567 (11.5) |  |  |
| 2016 | 8011 (10.3) | 7303 (11.1) | 2606 (11.6) |  |  |
| 2017 | 5533 (7.1) | 5124 (7.8) | 1819 (8.1) |  |  |
| 2018 | 4970 (6.4) | 4617 (7.0) | 1634 (7.3) |  |  |

Abbreviations: ADL, activities of daily living; ADRD, Alzheimer disease and related dementia; BPSD, behavioral and psychological symptoms of dementia; CNS, central nervous system; ED, emergency department; IPTW, inverse probability of treatment weighting; MME, morphine milligram equivalent; PHQ-9, Patient Health Questionnaire-9; PRN, as needed; SDiff, standardized difference.

^a^ Clinical conditions and health care use were measured in the year and other characteristics were measured in the 6 months before the index date (ie, opioid discontinuation for discontinuers and frequency-matched date for continuers)

^b^ Covariates with SDiff >0.100 represent meaningful differences between case and control groups.

^c^ A patient can contribute to more than 1 episode.

^d^Included Asian, Hispanic, Native American, and Pacific Islander.

**Table A.5.** Quarterly Associations of Discontinuing Long-term Opioid Therapy, Defined as a Gap of ≥90 Consecutive Days, With Worsening Clinical Outcomes from Baseline to Follow-Up

|  | **Discontinuing vs continuing LTOT** | | | | **Interaction of quarter time × LTOT discontinuation** | |
| --- | --- | --- | --- | --- | --- | --- |
| **Outcome by quarter ^a^** | **Crude OR**  **(95% CI)** | ***P* value** | **Adjusted OR^b^**  **(95% CI)** | ***P* value** | **Adjusted OR^b^**  **(95% CI)** | ***P* value** |
| ***Worsening pain*** |  |  |  |  |  |  |
| Quarter 1 | 0.78 (0.76-0.80) | <.001 | 0.86 (0.82-0.89) | <.001 | 0.91 (0.90-0.93) | <.001 |
| Quarter 2 | 0.71 (0.70-0.73) | <.001 | 0.82 (0.79-0.85) | <.001 |  |  |
| Quarter 3 | 0.49 (0.47-0.51) | <.001 | 0.69 (0.65-0.73) | <.001 |  |  |
| Quarter 4 | 0.47 (0.45-0.48) | <.001 | 0.66 (0.63-0.70) | <.001 |  |  |
| Quarter 5 | 0.44 (0.42-0.46) | <.001 | 0.65 (0.61-0.70) | <.001 |  |  |
| Quarter 6 | 0.48 (0.45-0.50) | <.001 | 0.69 (0.64-0.74) | <.001 |  |  |
| Quarter 7 | 0.44 (0.42-0.46) | <.001 | 0.63 (0.59-0.68) | <.001 |  |  |
| Quarter 8 | 0.41 (0.40-0.43) | <.001 | 0.60 (0.56-0.65) | <.001 |  |  |
| ***Worsening physical function*** | |  |  |  |  |  |
| Quarter 1 | 1.16 (1.13-1.19) | <.001 | 1.19 (1.15-1.23) | <.001 | 0.94 (0.93-0.95) | <.001 |
| Quarter 2 | 1.07 (1.05-1.10) | <.001 | 1.11 (1.07-1.15) | <.001 |  |  |
| Quarter 3 | 1.00 (0.97-1.03) | 0.836 | 0.96 (0.92-1.01) | 0.085 |  |  |
| Quarter 4 | 0.98 (0.96-1.01) | 0.300 | 0.97 (0.93-1.01) | 0.133 |  |  |
| Quarter 5 | 1.04 (1.00-1.09) | 0.035 | 0.99 (0.93-1.05) | 0.720 |  |  |
| Quarter 6 | 1.01 (0.98-1.05) | 0.450 | 0.95 (0.90-1.01) | 0.075 |  |  |
| Quarter 7 | 1.04 (1.00-1.08) | 0.046 | 0.96 (0.91-1.02) | 0.202 |  |  |
| Quarter 8 | 1.03 (0.99-1.07) | 0.118 | 0.96 (0.90-1.01) | 0.113 |  |  |
| ***Worsening depressive symptoms*** | |  |  |  |  |  |
| Quarter 1 | 1.05 (1.02-1.08) | <.001 | 1.11 (1.06-1.14) | <.001 | 0.96 (0.94-0.97) | <.001 |
| Quarter 2 | 0.95 (0.93-0.98) | <.001 | 1.00 (0.96-1.03) | 0.916 |  |  |
| Quarter 3 | 0.88 (0.86-0.91) | <.001 | 0.95 (0.90-0.99) | 0.016 |  |  |
| Quarter 4 | 0.90 (0.87-0.93) | <.001 | 0.95 (0.91-0.99) | 0.038 |  |  |
| Quarter 5 | 0.88 (0.85-0.92) | <.001 | 0.96 (0.90-1.02) | 0.209 |  |  |
| Quarter 6 | 0.87 (0.83-0.90) | <.001 | 0.91 (0.86-0.97) | 0.003 |  |  |
| Quarter 7 | 0.88 (0.85 -0.92) | <.001 | 0.96 (0.90-1.02) | 0.152 |  |  |
| Quarter 8 | 0.85 (0.82-0.88) | <.001 | 0.89 (0.84-0.94) | <.001 |  |  |

Abbreviations: LTOT, long-term opioid therapy; OR, odds ratio.

^a^ Data for quarters 1 to 4 were derived from the eligible sample with ≥1 year of follow-up; data for quarters 5 to 8 with ≥2 years of follow-up.

^b^ Adjusted baseline variables via the inverse probability of treatment weighting and the time-varying confounders (including use of adjuvant analgesics, use of as needed pain medications, use of any pain intervention, use of central nervous system medications, and dementia severity) as covariates.

**Table A.6.** Quarterly Associations of Discontinuing Long-term Opioid Therapy With Worsening Clinical Outcomes, Stratified by Residents With Mild or Moderate ADRD at Baseline

|  | **Continuing vs Discontinuing LTOT** | | | | | | | |
| --- | --- | --- | --- | --- | --- | --- | --- | --- |
|  | **Residents with mild ADRD at baseline** | | | | **Residents with moderate ADRD at baseline** | | | |
| **Outcomes by Quarters ^a^** | **Crude OR**  **(95% CI)** | ***P* value** | **Adjusted OR^b^**  **(95% CI)** | ***P* value** | **Crude OR**  **(95% CI)** | ***P* value** | **Adjusted OR^b^**  **(95% CI)** | ***P* value** |
| ***Worsening pain*** |  |  |  |  |  |  |  |  |
| Quarter 1 | 0.88 (0.85-0.90) | <.001 | 0.88 (0.85-0.92) | <.001 | 0.98 (0.93-1.02) | 0.319 | 0.92 (0.87-0.98) | 0.011 |
| Quarter 2 | 0.80 (0.78-0.82) | <.001 | 0.84 (0.81-0.87) | <.001 | 0.89 (0.85-0.93) | <.001 | 0.90 (0.85-0.96) | <.001 |
| Quarter 3 | 0.58 (0.56-0.60) | <.001 | 0.71 (0.68-0.75) | <.001 | 0.63 (0.59-0.67) | <.001 | 0.76(0.70-0.83) | <.001 |
| Quarter 4 | 0.57 (0.55-0.59) | <.001 | 0.70 (0.66-0.73) | <.001 | 0.61 (0.57-0.64) | <.001 | 0.75 (0.69-0.82) | <.001 |
| Quarter 5 | 0.56 (0.54-0.59) | <.001 | 0.71 (0.66-0.76) | <.001 | 0.58 (0.53-0.63) | <.001 | 0.69 (0.62-0.78) | <.001 |
| Quarter 6 | 0.58 (0.55-0.61) | <.001 | 0.73 (0.68-0.78) | <.001 | 0.61 (0.56-0.66) | <.001 | 0.78 (0.69-0.87) | <.001 |
| Quarter 7 | 0.55 (0.53-0.58) | <.001 | 0.69 (0.65-0.74) | <.001 | 0.55 (0.50-0.59) | <.001 | 0.68 (0.61-0.77) | <.001 |
| Quarter 8 | 0.51 (0.49-0.54) | <.001 | 0.64 (0.60-0.68) | <.001 | 0.52 (0.49-0.57) | <.001 | 0.67 (0.60-0.75) | <.001 |
| ***Worsening physical function*** | |  |  |  |  |  |  |  |
| Quarter 1 | 1.25 (1.21-1.28) | <.001 | 1.27 (1.23-1.31) | <.001 | 1.05 (1.01-1.10) | 0.011 | 1.14 (1.09-1.20) | <.001 |
| Quarter 2 | 1.11 (1.08-1.14) | <.001 | 1.14 (1.10-1.18) | <.001 | 0.98 (0.94-1.02) | 0.247 | 1.09 (1.04-1.15) | <.001 |
| Quarter 3 | 1.06 (1.02-1.09) | <.001 | 1.05 (1.01-1.10) | 0.041 | 0.90 (0.86-0.94) | <.001 | 0.97 (0.91-1.03) | 0.361 |
| Quarter 4 | 1.07 (1.03-1.10) | <.001 | 1.04 (0.99-1.08) | 0.108 | 0.87 (0.84-0.92) | <.001 | 0.95 (0.89-1.01) | 0.130 |
| Quarter 5 | 1.11 (1.07-1.16) | <.001 | 1.06 (0.99-1.13) | 0.054 | 0.90 (0.85-0.96) | <.001 | 0.92 (0.85-1.01) | 0.077 |
| Quarter 6 | 1.08 (1.04-1.13) | <.001 | 1.06 (0.99-1.12) | 0.068 | 0.93 (0.87-0.99) | 0.016 | 0.97 (0.89-1.06) | 0.520 |
| Quarter 7 | 1.10 (1.06-1.15) | <.001 | 1.06 (0.99-1.12) | 0.054 | 0.93 (0.87-0.98) | 0.012 | 0.96 (0.88-1.05) | 0.349 |
| Quarter 8 | 1.06 (1.02-1.10) | 0.005 | 1.03 (0.97-1.09) | 0.301 | 0.94 (0.88-0.99) | 0.028 | 0.97 (0.89-1.06) | 0.485 |
| ***Worsening depressive symptoms*** | |  |  |  |  |  |  |  |
| Quarter 1 | 1.10 (1.06-1.13) | <.001 | 1.14 (1.11-1.18) | <.001 | 1.02 (0.97-1.06) | 0.427 | 1.05 (0.99-1.11) | 0.096 |
| Quarter 2 | 0.98 (0.96-1.01) | 0.245 | 1.02 (0.98-1.06) | 0.266 | 0.92 (0.89-0.96) | <.001 | 0.95 (0.90-1.00) | 0.055 |
| Quarter 3 | 0.91 (0.88-0.95) | <.001 | 0.96 (0.92-1.01) | 0.099 | 0.88 (0.84-0.93) | <.001 | 0.97 (0.91-1.04) | 0.380 |
| Quarter 4 | 0.92 (0.88-0.05) | <.001 | 0.94 (0.90-0.98) | 0.007 | 0.93 (0.89-0.97) | 0.002 | 0.98 (0.92-1.04) | 0.583 |
| Quarter 5 | 0.89 (0.85-0.93) | <.001 | 0.93 (0.87-0.99) | 0.028 | 0.93 (0.87-0.99) | 0.020 | 1.00 (0.91-1.09) | 0.954 |
| Quarter 6 | 0.88 (0.84-0.92) | <.001 | 0.91 (0.85-0.97) | 0.002 | 0.93 (0.87-0.98) | 0.014 | 1.00 (0.92-1.09) | 0.946 |
| Quarter 7 | 0.91 (0.88-0.95) | <.001 | 0.97 (0.91-.103) | 0.312 | 0.87 (0.82-0.93) | <.001 | 0.92 (0.85-1.01) | 0.073 |
| Quarter 8 | 0.87 (0.83-0.90) | <.001 | 0.91 (0.86-0.97) | 0.002 | 0.87 (0.82-0.92) | <.001 | 0.91 (0.84-0.99) | 0.029 |

Abbreviations: ADRD, Alzheimer disease and related dementias; LTOT, long-term opioid therapy; OR, odds ratio.

^a^ Data for quarters 1 to 4 were derived from the eligible sample with ≥1 year of follow-up; data for quarters 5 to 8, with ≥2 years of follow-up.

^b^ Adjusted baseline variables (excluding baseline dementia severity) via the inverse probability of treatment weighting and the time-varying confounders (including use of adjuvant analgesics, use of as needed pain medications, use of any pain intervention, use of central nervous system medications, and dementia severity) as covariates.

**Table A.7.** Quarterly Associations of Discontinuing Long-term Opioid Therapy With Worsening Clinical Outcomes, Stratified by Residents With Types of Pain Conditions at Baseline

|  | **Continuing vs Discontinuing LTOT** | | | | | | | | | | | |
| --- | --- | --- | --- | --- | --- | --- | --- | --- | --- | --- | --- | --- |
|  | **Residents with musculoskeletal pain at baseline** | | | | **Residents with neuropathic pain at baseline** | | | | **Residents with idiopathic pain at baseline** | | | |
| **Outcomes by Quarters ^a^** | **Crude OR**  **(95% CI)** | ***P* value** | **Adjusted OR^b^**  **(95% CI)** | ***P* value** | **Crude OR**  **(95% CI)** | ***P* value** | **Adjusted OR^b^**  **(95% CI)** | ***P* value** | **Crude OR**  **(95% CI)** | ***P* value** | **Adjusted OR^b^**  **(95% CI)** | ***P* value** |
| ***Worsening pain*** | |  |  |  |  |  |  |  |  |  |  |  |
| Quarter 1 | 0.90 (0.88-0.93) | <.001 | 0.90 (0.88-0.93) | <.001 | 0.87 (0.83-0.90) | <.001 | 0.88 (0.84-0.92) | <.001 | 0.87 (0.84-0.90) | <.001 | 0.89 (0.85-0.93) | <.001 |
| Quarter 2 | 0.82 (0.81-0.84) | <.001 | 0.86 (0.83-0.89) | <.001 | 0.80 (0.77-0.83) | <.001 | 0.84 (0.80-0.88) | <.001 | 0.81 (0.78-0.84) | <.001 | 0.86 (0.81-0.90) | <.001 |
| Quarter 3 | 0.58 (0.56-0.60) | <.001 | 0.72 (0.69-0.76) | <.001 | 0.57 (0.54-0.60) | <.001 | 0.70 (0.65-0.75) | <.001 | 0.58 (0.55-0.61) | <.001 | 0.71 (0.66-0.76) | <.001 |
| Quarter 4 | 0.57 (0.55-0.58) | <.001 | 0.71 (0.68-0.74) | <.001 | 0.55 (0.52-0.57) | <.001 | 0.67 (0.63-0.72) | <.001 | 0.54 (0.52-0.57) | <.001 | 0.67 (0.63-0.72) | <.001 |
| Quarter 5 | 0.54 (0.52-0.57) | <.001 | 0.70 (0.66-0.75) | <.001 | 0.51 (0.47-0.54) | <.001 | 0.64 (0.58-0.71) | <.001 | 0.51 (0.48-0.55) | <.001 | 0.66 (0.60-0.72) | <.001 |
| Quarter 6 | 0.57 (0.55-0.59) | <.001 | 0.73 (0.69-0.77) | <.001 | 0.54 (0.51-0.58) | <.001 | 0.72 (0.66-0.79) | <.001 | 0.57 (0.53-0.61) | <.001 | 0.76 (0.70-0.84) | <.001 |
| Quarter 7 | 0.52 (0.50-0.54) | <.001 | 0.68 (0.64-0.72) | <.001 | 0.53 (0.49-0.56) | <.001 | 0.70 (0.64-0.77) | <.001 | 0.53 (0.49-0.56) | <.001 | 0.68 (0.62-0.75) | <.001 |
| Quarter 8 | 0.50 (0.48-0.52) | <.001 | 0.65 (0.61-0.68) | <.001 | 0.49 (0.46-0.52) | <.001 | 0.64 (0.59-0.70) | <.001 | 0.50 (0.47-0.53) | <.001 | 0.64 (0.59-0.70) | <.001 |
| ***Worsening physical function*** | |  |  |  |  |  |  |  |  |  |  |  |
| Quarter 1 | 1.18 (1.16-1.21) | <.001 | 1.22 (1.19-1.26) | <.001 | 1.27 (1.22-1.32) | <.001 | 1.28 (1.22-1.34) | <.001 | 1.27 (1.22-1.32) | <.001 | 1.28 (1.22-1.34) | <.001 |
| Quarter 2 | 1.07 (1.04-1.09) | <.001 | 1.12 (1.09-1.15) | <.001 | 1.11 (1.07-1.15) | <.001 | 1.14 (1.09-1.20) | <.001 | 1.11 (1.07-1.15) | <.001 | 1.15 (1.10-1.20) | <.001 |
| Quarter 3 | 1.01 (0.99-1.04) | <.001 | 1.01 (0.98-1.05) | 0.439 | 1.05 (1.00-1.10) | 0.061 | 1.03 (0.97-1.09) | 0.384 | 1.06 (1.01-1.11) | 0.061 | 1.04 (0.98-1.10) | 0.219 |
| Quarter 4 | 1.01 (0.99-1.04) | <.001 | 1.01 (0.97-1.05) | 0.618 | 1.07 (1.02-1.12) | 0.003 | 1.04 (0.98-1.11) | 0.153 | 1.07 (1.02-1.12) | 0.003 | 1.04 (0.98-1.11) | 0.240 |
| Quarter 5 | 1.03 (0.99-1.07) | 0.097 | 1.02 (0.97-1.07) | 0.401 | 1.03 (0.97-1.10) | 0.329 | 1.02 (0.94-1.11) | 0.659 | 1.07 (1.01-1.14) | 0.028 | 1.07 (0.99-1.17) | 0.09 |
| Quarter 6 | 1.03 (0.99-1.06) | 0.101 | 1.03 (0.98-1.08) | 0.302 | 1.03 (0.97-1.10) | 0.269 | 1.02 (0.94-1.11) | 0.588 | 1.04 (0.98-1.11) | 0.159 | 1.04 (0.96-1.12) | 0.376 |
| Quarter 7 | 1.05 (1.02-1.09) | 0.004 | 1.04 (0.99-1.09) | 0.159 | 1.07 (1.01-1.14) | 0.020 | 1.06 (0.98-1.15) | 0.144 | 1.09 (1.03-1.16) | 0.003 | 1.12 (1.03-1.21) | 0.008 |
| Quarter 8 | 1.04 (1.00-1.07) | 0.043 | 1.02 (0.97-1.06) | 0.535 | 1.07 (1.01-1.14) | 0.020 | 1.05 (0.97-1.13) | 0.263 | 1.07 (1.01-1.14) | 0.018 | 1.04 (0.96-1.13) | 0.315 |
| ***Worsening depressive symptoms*** | |  |  |  |  |  |  |  |  |  |  |  |
| Quarter 1 | 1.07 (1.04-1.10) | <.001 | 1.11 (1.08-1.15) | <.001 | 1.09 (1.05-1.14) | <.001 | 1.12 (1.06-1.17) | <.001 | 1.10 (1.06-1.15) | <.001 | 1.13 (1.07-1.18) | <.001 |
| Quarter 2 | 0.96 (0.94-0.98) | <.001 | 1.00 (0.97-1.03) | 0.926 | 0.98 (0.94-1.02) | 0.365 | 1.04 (0.99-1.09) | 0.141 | 0.98 (0.94-1.02) | 0.415 | 1.03 (0.98-1.08) | 0.219 |
| Quarter 3 | 0.90 (0.87-0.93) | <.001 | 0.96 (0.92-0.99) | 0.038 | 0.88 (0.84-0.93) | <.001 | 0.94 (0.88-0.99) | 0.047 | 0.88 (0.84-0.93) | <.001 | 0.94 (0.88-1.00) | 0.043 |
| Quarter 4 | 0.92 (0.89-0.95) | <.001 | 0.95 (0.92-0.99) | 0.011 | 0.93 (0.89-0.98) | 0.003 | 0.96 (0.90-1.02) | 0.171 | 0.95 (0.90-0.99) | 0.027 | 0.98 (0.92-1.04) | 0.431 |
| Quarter 5 | 0.89 (0.86-0.93) | <.001 | 0.95 (0.90-1.00) | 0.069 | 0.87 (0.81-0.93) | <.001 | 0.92 (0.84-1.01) | 0.065 | 0.87 (0.81-0.93) | <.001 | 0.92 (0.84-1.00) | 0.057 |
| Quarter 6 | 0.89 (0.85-0.92) | <.001 | 0.93 (0.89-0.98) | 0.005 | 0.87 (0.82-0.93) | <.001 | 0.88 (0.81-0.96) | 0.003 | 0.86 (0.81-0.92) | 0.415 | 0.87 (0.80-0.95) | 0.002 |
| Quarter 7 | 0.89 (0.86-0.92) | <.001 | 0.95 (0.90-0.99) | 0.039 | 0.89 (0.84-0.95) | <.001 | 0.93 (0.86-1.01) | 0.095 | 0.86 (0.81-0.92) | <.001 | 0.92 (0.85-1.00) | 0.061 |
| Quarter 8 | 0.86 (0.83-0.89) | <.001 | 0.91 (0.86-0.95) | <.001 | 0.89 (0.84-0.95) | <.001 | 0.93 (0.86-1.00) | 0.064 | 0.90 (0.84-0.95) | <.001 | 0.94 (0.87-1.02) | 0.121 |

Abbreviations: ADRD, Alzheimer disease and related dementias; LTOT, long-term opioid therapy; OR, odds ratio.

^a^ Data for quarters 1 to 4 were derived from the eligible sample with ≥1 year of follow-up; data for quarters 5 to 8, with ≥2 years of follow-up.

^b^ Adjusted baseline variables via the inverse probability of treatment weighting and the time-varying confounders (including use of adjuvant analgesics, use of as needed pain medications, use of any pain intervention, use of central nervous system medications, and dementia severity) as covariates.

**Table A.8.** Quarterly Associations of Discontinuing Long-term Opioid Therapy With Worsening Clinical Outcomes, Stratified by Race and Ethnicity

|  | **Continuing vs Discontinuing LTOT** | | | | | | | | | | | |
| --- | --- | --- | --- | --- | --- | --- | --- | --- | --- | --- | --- | --- |
|  | **Residents who are White** | | | | **Residents who are Black** | | | | **Residents who are other race ^c^** | | | |
| **Outcomes by Quarters ^a^** | **Crude OR**  **(95% CI)** | ***P* value** | **Adjusted OR^b^**  **(95% CI)** | ***P* value** | **Crude OR**  **(95% CI)** | ***P* value** | **Adjusted OR^b^**  **(95% CI)** | ***P* value** | **Crude OR**  **(95% CI)** | ***P* value** | **Adjusted OR^b^**  **(95% CI)** | ***P* value** |
| ***Worsening pain*** | |  |  |  |  |  |  |  |  |  |  |  |
| Quarter 1 | 0.90 (0.88-0.93) | <.001 | 0.89 (0.86-0.92) | <.001 | 0.98 (0.91-1.04) | 0.468 | 0.92 (0.85-1.01) | 0.068 | 0.94 (0.86-1.03) | 0.195 | 0.98 (0.87-1.11) | 0.727 |
| Quarter 2 | 0.84 (0.81-0.86) | <.001 | 0.86 (0.83-0.89) | <.001 | 0.87 (0.81-0.93) | <.001 | 0.87 (0.80-0.95) | 0.002 | 0.80 (0.73-0.88) | <.001 | 0.86 (0.76-0.97) | 0.014 |
| Quarter 3 | 0.59 (0.57-0.61) | <.001 | 0.73 (0.69-0.76) | <.001 | 0.61 (0.56-0.67) | <.001 | 0.74 (0.66-0.83) | <.001 | 0.53 (0.47-0.59) | <.001 | 0.69 (0.59-0.81) | <.001 |
| Quarter 4 | 0.58 (0.56-0.60) | <.001 | 0.72 (0.68-0.75) | <.001 | 0.59 (0.54-0.64) | <.001 | 0.72 (0.65-0.81) | <.001 | 0.50 (0.44-0.56) | <.001 | 0.63 (0.54-0.73) | <.001 |
| Quarter 5 | 0.55 (0.53-0.58) | <.001 | 0.71 (0.66-0.76) | <.001 | 0.56 (0.50-0.63) | <.001 | 0.70 (0.60-0.82) | <.001 | 0.52 (0.45-0.61) | <.001 | 0.66 (0.54-0.81) | <.001 |
| Quarter 6 | 0.58 (0.55-0.60) | <.001 | 0.74 (0.69-0.97) | <.001 | 0.59 (0.53-0.66) | <.001 | 0.73 (0.63-0.84) | <.001 | 0.54 (0.47-0.63) | <.001 | 0.73 (0.60-0.88) | 0.001 |
| Quarter 7 | 0.53 (0.51-0.56) | <.001 | 0.69 (0.65-0.74) | <.001 | 0.57 (0.51-0.63) | <.001 | 0.67 (0.58-0.78) | <.001 | 0.47 (0.41-0.55) | <.001 | 0.61 (0.49-0.74) | <.001 |
| Quarter 8 | 0.51 (0.49-0.53) | <.001 | 0.65 (0.61-0.69) | <.001 | 0.53 (0.48-0.59) | <.001 | 0.63 (0.54-0.72) | <.001 | 0.50 (0.44-0.58) | <.001 | 0.66 (0.55-0.80) | <.001 |
| ***Worsening physical function*** | |  |  |  |  |  |  |  |  |  |  |  |
| Quarter 1 | 1.17 (1.15-1.29) | <.001 | 1.23 (1.19-1.27) | <.001 | 1.18 (1.11-1.26) | <.001 | 1.20 (1.12-1.30) | <.001 | 1.22 (1.12-1.32) | <.001 | 1.23 (1.11-1.36) | <.001 |
| Quarter 2 | 1.06 (1.03-1.08) | <.001 | 1.12 (1.09-1.16) | <.001 | 1.09 (1.02-1.16) | 0.080 | 1.10 (1.02-1.19) | 0.016 | 1.16 (1.07-1.26) | <.001 | 1.20 (1.08-1.33) | <.001 |
| Quarter 3 | 1.00 (0.97-1.03) | 0.870 | 1.01 (0.97-1.05) | 0.543 | 1.08 (1.00-1.16) | 0.046 | 1.06 (0.97-1.17) | 0.192 | 1.08 (0.98-1.19) | 0.115 | 1.04 (0.92-1.18) | 0.500 |
| Quarter 4 | 1.02 (0.99-1.05) | 0.252 | 1.02 (0.98-1.06) | 0.354 | 1.04 (0.97-1.12) | 0.261 | 0.99 (0.91-1.09) | 0.915 | 0.98 (0.89-1.07) | 0.597 | 0.91 (0.81-1.03) | 0.148 |
| Quarter 5 | 1.02 (0.98-1.06) | 0.299 | 1.01 (0.96-1.07) | 0.708 | 1.07 (0.98-1.17) | 0.151 | 1.07 (0.94-1.21) | 0.304 | 1.06 (0.94-1.20) | 0.335 | 1.05 (0.88-1.24) | 0.599 |
| Quarter 6 | 1.02 (0.98-1.06) | 0.336 | 1.00 (0.94-1.05) | 0.904 | 1.08 (0.99-1.18) | 0.087 | 1.14 (1.01-1.28) | 0.036 | 1.11 (0.99-1.25) | 0.087 | 1.14 (0.97-1.34) | 0.124 |
| Quarter 7 | 1.04 (1.00-1.08) | 0.027 | 1.01 (0.96-1.07) | 0.666 | 1.11 (1.02-1.21) | 0.017 | 1.14 (1.01-1.28) | 0.032 | 1.11 (0.99-1.25) | 0.086 | 1.14 (0.97-1.33) | 0.113 |
| Quarter 8 | 1.03 (1.00-1.07) | 0.075 | 1.00 (0.95-1.06) | 0.954 | 1.05 (0.97-1.15) | 0.248 | 1.01 (0.90-1.14) | 0.804 | 1.16 (1.03-1.30) | 0.012 | 1.17 (1.00-1.37) | 0.053 |
| ***Worsening depressive symptoms*** | |  |  |  |  |  |  |  |  |  |  |  |
| Quarter 1 | 1.09 (1.06-1.12) | <.001 | 1.12 (1.08-1.16) | <.001 | 1.09 (1.01-1.17) | 0.019 | 1.10 (1.01-1.20) | 0.036 | 1.05 (0.95-1.15) | 0.355 | 1.06 (0.94-1.20) | 0.331 |
| Quarter 2 | 0.96 (0.94-0.99) | 0.007 | 0.98 (0.95-1.02) | 0.344 | 1.10 (1.02-1.17) | 0.010 | 1.12 (1.03-1.22) | 0.011 | 0.96 (0.88-1.06) | 0.432 | 1.02 (0.91-1.15) | 0.709 |
| Quarter 3 | 0.91 (0.89-0.94) | <.001 | 0.96 (0.92-1.00) | 0.070 | 0.97 (0.89-1.06) | 0.496 | 0.95 (0.86-1.06) | 0.388 | 0.86 (0.77-0.96) | 0.008 | 0.95 (0.83-1.09) | 0.498 |
| Quarter 4 | 0.94 (0.91-0.97) | <.001 | 0.95 (0.91-0.99) | 0.027 | 0.99 (0.91-1.07) | 0.720 | 0.97 (0.88-1.07) | 0.549 | 0.83 (0.75-0.92) | <.001 | 0.85 (0.74-0.97) | 0.016 |
| Quarter 5 | 0.90 (0.87-0.94) | <.001 | 0.96 (0.90-1.02) | 0.169 | 0.99 (0.89-1.10) | 0.841 | 0.93 (0.81-1.07) | 0.299 | 0.87 (0.76-1.01) | 0.059 | 0.92 (0.77-1.11) | 0.405 |
| Quarter 6 | 0.91 (0.87-0.94) | <.001 | 0.94 (0.88-0.99) | 0.021 | 0.93 (0.84-1.03) | 0.151 | 0.95 (0.84-1.08) | 0.439 | 0.83 (0.73-0.95) | 0.008 | 0.88 (0.74-1.06) | 0.172 |
| Quarter 7 | 0.90 (0.86-0.93) | <.001 | 0.95 (0.90-1.00) | 0.058 | 1.01 (0.92-1.11) | 0.830 | 1.02 (0.90-1.15) | 0.793 | 0.94 (0.82-1.07) | 0.334 | 1.01 (0.85-1.19) | 0.935 |
| Quarter 8 | 0.88 (0.85-0.92) | <.001 | 0.91 (0.86-0.96) | <.001 | 0.92 (0.84-1.01) | 0.097 | 0.93 (0.82-1.05) | 0.236 | 0.83 (0.73-0.94) | 0.004 | 0.95 (0.80-1.13) | 0.572 |

Abbreviations: ADRD, Alzheimer disease and related dementias; LTOT, long-term opioid therapy; OR, odds ratio.

^a^ Data for quarters 1 to 4 were derived from the eligible sample with ≥1 year of follow-up; data for quarters 5 to 8, with ≥2 years of follow-up.

^b^ Adjusted baseline variables (except for race/ethnicity) via the inverse probability of treatment weighting and the time-varying confounders (including use of adjuvant analgesics, use of as needed pain medications, use of any pain intervention, use of central nervous system medications, and dementia severity) as covariates.

^c^ Included Asian, Hispanic, Native American, and Pacific Islander

**Table A.9.** Quarterly Associations of Discontinuing Long-term Opioid Therapy With Worsening Clinical Outcomes, Stratified According to No-to-Mild or Moderate-to-Severe Pain at Baseline

|  | **Continuing vs Discontinuing LTOT** | | | | | | | |
| --- | --- | --- | --- | --- | --- | --- | --- | --- |
|  | **Residents with no/mild pain at baseline** | | | | **Residents with moderate/severe pain at baseline** | | | |
| **Outcomes by quarter ^a^** | **Crude OR**  **(95% CI)** | ***P* value** | **Adjusted OR^b^**  **(95% CI)** | ***P* value** | **Crude OR**  **(95% CI)** | ***P* value** | **Adjusted OR^b^**  **(95% CI)** | ***P* value** |
| ***Worsening pain*** |  |  |  |  |  |  |  |  |
| Quarter 1 | 1.07 (1.04-1.10) | <.001 | 1.03 (0.99-1.08) | 0.111 | 0.69 (0.66-0.72) | <.001 | 0.75 (0.71-0.79) | <.001 |
| Quarter 2 | 0.90 (0.90-0.96) | <.001 | 0.93 (0.89-0.97) | <.001 | 0.67 (0.65-0.70) | <.001 | 0.75 (0.72-0.79) | <.001 |
| Quarter 3 | 0.66 (0.63-0.68) | <.001 | 0.77 (0.73-0.82) | <.001 | 0.52 (0.49-0.54) | <.001 | 0.67(0.63-0.72) | <.001 |
| Quarter 4 | 0.63 (0.61-0.66) | <.001 | 0.74 (0.70-0.78) | <.001 | 0.51 (0.49-0.54) | <.001 | 0.67 (0.62-0.72) | <.001 |
| Quarter 5 | 0.59 (0.56-0.62) | <.001 | 0.70 (0.65-0.75) | <.001 | 0.53 (0.50-0.57) | <.001 | 0.72 (0.65-0.79) | <.001 |
| Quarter 6 | 0.62 (0.59-0.65) | <.001 | 0.72 (0.67-0.77) | <.001 | 0.54 (0.51-0.58) | <.001 | 0.74 (0.67-0.81) | <.001 |
| Quarter 7 | 0.56 (0.54-0.59) | <.001 | 0.68 (0.63-0.73) | <.001 | 0.53 (0.50-0.57) | <.001 | 0.67 (0.61-0.74) | <.001 |
| Quarter 8 | 0.54 (0.51-0.56) | <.001 | 0.66 (0.61-0.70) | <.001 | 0.47 (0.44-0.51) | <.001 | 0.60 (0.55-0.66) | <.001 |
| ***Worsening physical function*** | |  |  |  |  |  |  |  |
| Quarter 1 | 1.16 (1.13-1.20) | <.001 | 1.21 (1.16-1.25) | <.001 | 1.22 (1.17-1.27) | <.001 | 1.27 (1.22-1.33) | <.001 |
| Quarter 2 | 1.06 (1.04-1.09) | <.001 | 1.14 (1.10-1.18) | <.001 | 1.08 (1.04-1.12) | <.001 | 1.10 (1.05-1.16) | <.001 |
| Quarter 3 | 1.02 (0.99-1.05) | 0.249 | 1.05 (1.01-1.10) | 0.021 | 0.99 (0.94-1.04) | 0.669 | 0.95 (0.89-1.02) | 0.136 |
| Quarter 4 | 1.01 (0.98-1.04) | 0.607 | 1.00 (0.96-1.04) | 0.972 | 0.99 (0.94-1.04) | 0.700 | 1.03 (0.97-1.10) | 0.365 |
| Quarter 5 | 1.05 (1.01-1.10) | 0.024 | 1.00 (0.95-1.07) | 0.893 | 1.04 (0.98-1.11) | 0.237 | 1.03 (0.94-1.12) | 0.575 |
| Quarter 6 | 1.06 (1.02-1.11) | 0.004 | 1.02 (0.96-1.08) | 0.509 | 0.99 (0.93-1.05) | 0.746 | 1.04 (0.96-1.13) | 0.357 |
| Quarter 7 | 1.05 (1.01-1.10) | 0.181 | 0.98 (0.93-1.04) | 0.594 | 1.05 (0.99-1.12) | 0.115 | 1.11 (1.02-1.20) | 0.017 |
| Quarter 8 | 1.03 (0.99-1.08) | 0.125 | 0.99 (0.93-1.05) | 0.676 | 1.02 (0.96-1.08) | 0.523 | 1.05 (0.97-1.14) | 0.228 |
| ***Worsening depressive symptoms*** | |  |  |  |  |  |  |  |
| Quarter 1 | 1.10 (1.06-1.13) | <.001 | 1.13 (1.08-1.17) | <.001 | 1.02 (0.98-1.07) | 0.237 | 1.09 (1.04-1.14) | <.001 |
| Quarter 2 | 0.97 (0.94-1.00) | 0.072 | 1.00 (0.97-1.04) | 0.799 | 0.94 (0.90-0.98) | 0.002 | 0.99 (0.94-1.04) | 0.622 |
| Quarter 3 | 0.92 (0.89-0.95) | <.001 | 0.96 (0.92-1.00) | 0.076 | 0.88 (0.84-0.93) | <.001 | 0.98 (0.92-1.05) | 0.644 |
| Quarter 4 | 0.94 (0.91-0.97) | <.001 | 0.96 (0.82-1.00) | 0.059 | 0.90 (0.86-0.94) | <.001 | 0.95 (0.89-1.01) | 0.122 |
| Quarter 5 | 0.94 (0.90-0.98) | 0.007 | 0.98 (0.92-1.04) | 0.543 | 0.85 (0.79-0.90) | <.001 | 0.90 (0.82-0.98) | 0.022 |
| Quarter 6 | 0.91 (0.87-0.95) | <.001 | 0.92 (0.87-0.98) | 0.008 | 0.87 (0.82-0.93) | <.001 | 0.98 (0.89-1.07) | 0.609 |
| Quarter 7 | 0.91 (0.87-0.95) | <.001 | 0.95 (0.90-.101) | 0.098 | 0.90 (0.84-0.96) | <.001 | 0.97 (0.89-1.05) | 0.425 |
| Quarter 8 | 0.89 (0.85-0.93) | <.001 | 0.92 (0.87-0.97) | 0.004 | 0.85 (0.80-0.90) | <.001 | 0.89 (0.82-0.97) | 0.007 |

Abbreviations: ADRD, Alzheimer disease and related dementias; LTOT, long-term opioid therapy; OR, odds ratio.

^a^ Data for quarters 1 to 4 were derived from the eligible sample with ≥1 year of follow-up; data for quarters 5 to 8, with ≥2 years of follow-up.

^b^ Adjusted baseline variables (excluding baseline pain) via the inverse probability of treatment weighting and the time-varying confounders (including use of adjuvant analgesics, use of as needed pain medications, use of any pain intervention, use of central nervous system medications, and dementia severity) as covariates.

**Table A.10.** Quarterly Associations of Discontinuing Long-term Opioid Therapy With Worsening Clinical Outcomes, Stratified According to No-to-Mild or Moderate-to-Severe Physical Function at Baseline

|  | **Continuing vs Discontinuing LTOT** | | | | | | | |
| --- | --- | --- | --- | --- | --- | --- | --- | --- |
|  | **Residents with no/mild physical function at baseline** | | | | **Residents with moderate/severe physical function at baseline** | | | |
| **Outcomes by quarter ^a^** | **Crude OR**  **(95% CI)** | ***P* value** | **Adjusted OR^b^**  **(95% CI)** | ***P* value** | **Crude OR**  **(95% CI)** | ***P* value** | **Adjusted OR^b^**  **(95% CI)** | ***P* value** |
| ***Worsening pain*** |  |  |  |  |  |  |  |  |
| Quarter 1 | 0.89 (0.85-0.92) | <.001 | 0.90 (0.86-0.95) | <.001 | 0.93 (0.90-0.96) | <.001 | 0.90 (0.86-0.93) | <.001 |
| Quarter 2 | 0.82 (0.79-0.85) | <.001 | 0.87 (0.83-0.92) | <.001 | 0.84 (0.82-0.87) | <.001 | 0.85 (0.82-0.89) | <.001 |
| Quarter 3 | 0.58 (0.55-0.61) | <.001 | 0.71 (0.66-0.76) | <.001 | 0.59 (0.57-0.62) | <.001 | 0.74(0.70-0.78) | <.001 |
| Quarter 4 | 0.57 (0.54-0.60) | <.001 | 0.73 (0.68-0.79) | <.001 | 0.57 (0.55-0.60) | <.001 | 0.70 (0.66-0.74) | <.001 |
| Quarter 5 | 0.55 (0.52-0.59) | <.001 | 0.70 (0.64-0.77) | <.001 | 0.56 (0.53-0.59) | <.001 | 0.70 (0.65-0.76) | <.001 |
| Quarter 6 | 0.57 (0.53-0.60) | <.001 | 0.72 (0.66-0.79) | <.001 | 0.59 (0.56-0.62) | <.001 | 0.75 (0.69-0.80) | <.001 |
| Quarter 7 | 0.53 (0.50-0.57) | <.001 | 0.68 (0.62-0.74) | <.001 | 0.55 (0.52-0.58) | <.001 | 0.69 (0.64-0.75) | <.001 |
| Quarter 8 | 0.50 (0.47-0.53) | <.001 | 0.64 (0.59-0.70) | <.001 | 0.51 (0.48-0.54) | <.001 | 0.65 (0.60-0.70) | <.001 |
| ***Worsening physical function*** | |  |  |  |  |  |  |  |
| Quarter 1 | 1.70 (1.64-1.77) | <.001 | 1.71 (1.63-1.79) | <.001 | 0.91 (0.88-0.94) | <.001 | 1.02 (0.98-1.05) | 0.378 |
| Quarter 2 | 1.43 (1.38-1.48) | <.001 | 1.41 (1.35-1.48) | <.001 | 0.87 (0.85-0.90) | <.001 | 0.98 (0.95-1.01) | 0.256 |
| Quarter 3 | 1.19 (1.14-1.25) | <.001 | 1.09 (1.03-1.16) | 0.005 | 0.91 (0.88-0.94) | <.001 | 0.98 (0.94-1.03) | 0.426 |
| Quarter 4 | 1.15 (1.10-1.20) | <.001 | 1.04 (0.98-1.10) | 0.228 | 0.93 (0.90-0.96) | <.001 | 0.99 (0.95-1.04) | 0.707 |
| Quarter 5 | 1.24 (1.17-1.31) | <.001 | 1.07 (0.99-1.17) | 0.102 | 0.95 (0.91-0.99) | 0.025 | 0.98 (0.92-1.04) | 0.520 |
| Quarter 6 | 1.24 (1.17-1.31) | <.001 | 1.11 (1.03-1.20) | 0.010 | 0.95 (0.91-0.99) | 0.018 | 0.98 (0.92-1.04) | 0.482 |
| Quarter 7 | 1.26 (1.19-1.33) | <.001 | 1.11 (1.02-1.20) | 0.012 | 0.97 (0.93-1.01) | 0.112 | 0.98 (0.92-1.04) | 0.543 |
| Quarter 8 | 1.18 (1.12-1.25) | <.001 | 1.03 (0.95-1.12) | 0.469 | 0.97 (0.93-1.01) | 0.183 | 1.00 (0.94-1.16) | 0.982 |
| ***Worsening depressive symptoms*** | |  |  |  |  |  |  |  |
| Quarter 1 | 1.12 (1.08-1.17) | <.001 | 1.16 (1.10-1.22) | <.001 | 1.04 (1.01-1.17) | 0.008 | 1.09 (1.05-1.13) | <.001 |
| Quarter 2 | 0.99 (0.95-1.03) | 0.704 | 1.03 (0.98-1.08) | 0.261 | 0.95 (0.92-0.97) | <.001 | 0.99 (0.95-1.02) | 0.470 |
| Quarter 3 | 0.94 (0.89-0.98) | 0.010 | 1.01 (0.95-1.08) | 0.776 | 0.88 (0.85-0.92) | <.001 | 0.93 (0.89-0.98) | 0.005 |
| Quarter 4 | 0.91 (0.87-0.96) | <.001 | 0.90 (0.85-0.96) | 0.002 | 0.92 (0.89-0.96) | <.001 | 0.98 (0.93-1.02) | 0.295 |
| Quarter 5 | 0.90 (0.84-0.96) | <.001 | 0.92 (0.85-1.01) | 0.065 | 0.90 (0.86-0.95) | <.001 | 0.97 (0.90-1.03) | 0.292 |
| Quarter 6 | 0.95 (0.90-1.01) | 0.083 | 1.00 (0.92-1.09) | 0.960 | 0.86 (0.82-0.90) | <.001 | 0.89 (0.83-0.95) | <.001 |
| Quarter 7 | 0.91 (0.86-0.96) | 0.001 | 0.95 (0.87-.102) | 0.166 | 0.89 (0.86-0.93) | <.001 | 0.96 (0.90-1.02) | 0.156 |
| Quarter 8 | 0.89 (0.84-0.94) | <.001 | 0.90 (0.83-0.97) | 0.007 | 0.86 (0.82-0.90) | <.001 | 0.91 (0.86-0.97) | 0.002 |

Abbreviations: ADRD, Alzheimer disease and related dementias; LTOT, long-term opioid therapy; OR, odds ratio.

^a^ Data for quarters 1 to 4 were derived from the eligible sample with ≥1 year of follow-up; data for quarters 5-8, with ≥2 years of follow-up.

^b^ Adjusted baseline variables (excluding baseline physical function) via the inverse probability of treatment weighting and the time-varying confounders (including use of adjuvant analgesics, use of as needed pain medications, use of any pain intervention, use of central nervous system medications, and dementia severity) as covariates.

**Table A.11.** Quarterly Associations of Discontinuing Long-term Opioid Therapy With Worsening Clinical Outcomes, Stratified According to No-to-Mild or Moderate-to-Severe Depressive Symptoms at Baseline

|  | **Continuing vs Discontinuing LTOT** | | | | | | | |
| --- | --- | --- | --- | --- | --- | --- | --- | --- |
|  | **Residents with no/mild depressive symptoms at baseline** | | | | **Residents with moderate/severe depressive symptoms at baseline** | | | |
| **Outcomes by quarter ^a^** | **Crude OR**  **(95% CI)** | ***P* value** | **Adjusted OR^b^**  **(95% CI)** | ***P* value** | **Crude OR**  **(95% CI)** | ***P* value** | **Adjusted OR^b^**  **(95% CI)** | ***P* value** |
| ***Worsening pain*** |  |  |  |  |  |  |  |  |
| Quarter 1 | 0.91 (0.89-0.93) | <.001 | 0.90 (0.87-0.93) | <.001 | 0.83 (0.75-0.91) | <.001 | 0.95 (0.84-1.07) | 0.387 |
| Quarter 2 | 0.823(0.81-0.85) | <.001 | 0.86 (0.83-0.89) | <.001 | 0.78 (0.71-0.86) | <.001 | 0.89 (0.79-1.01) | 0.066 |
| Quarter 3 | 0.58 (0.56-0.60) | <.001 | 0.72 (0.69-0.76) | <.001 | 0.60 (0.53-0.67) | <.001 | 0.80 (0.67-0.94) | 0.008 |
| Quarter 4 | 0.57 (0.55-0.59) | <.001 | 0.71 (0.68-0.74) | <.001 | 0.55 (0.49-0.62) | <.001 | 0.74 (0.62-0.88) | <.001 |
| Quarter 5 | 0.56 (0.53-0.58) | <.001 | 0.71 (0.67-0.75) | <.001 | 0.49 (0.42-0.58) | <.001 | 0.68 (0.54-0.87) | 0.002 |
| Quarter 6 | 0.58 (0.55-0.60) | <.001 | 0.74 (0.69-0.78) | <.001 | 0.56 (0.48-0.65) | <.001 | 0.75 (0.60-0.94) | 0.013 |
| Quarter 7 | 0.54 (0.52-0.56) | <.001 | 0.69 (0.65-0.73) | <.001 | 0.52 (0.44-0.61) | <.001 | 0.69 (0.55-0.86) | 0.001 |
| Quarter 8 | 0.50 (0.48-0.52) | <.001 | 0.64 (0.61-0.68) | <.001 | 0.51 (0.43-0.59) | <.001 | 0.67 (0.54-0.84) | <.001 |
| ***Worsening physical function*** | |  |  |  |  |  |  |  |
| Quarter 1 | 1.19 (1.16-1.22) | <.001 | 1.24(1.20-1.28) | <.001 | 1.04 (0.95-1.14) | 0.373 | 1.06 (0.95-1.19) | 0.293 |
| Quarter 2 | 1.08 (1.05-1.10) | <.001 | 1.13 (1.10-1.16) | <.001 | 0.95 (0.87-1.04) | 0.296 | 1.01 (0.90-1.13) | 0.928 |
| Quarter 3 | 1.02 (1.00-1.05) | 0.088 | 1.03 (0.99-1.07) | 0.147 | 0.90 (0.81-1.01) | 0..063 | 0.88 (0.76-1.02) | 0.093 |
| Quarter 4 | 1.01 (0.99-1.04) | 0.280 | 1.01 (0.97-1.05) | 0.662 | 0.98 (0.88-1.09) | 0.664 | 1.04 (0.89-1.21) | 0.610 |
| Quarter 5 | 1.06 (1.02-1.10) | 0.003 | 1.03 (0.98-1.08) | 0.305 | 1.00 (0.86-1.16) | 0.996 | 0.83 (0.66-1.05) | 0.125 |
| Quarter 6 | 1.06 (1.02-1.10) | 0.002 | 1.03 (0.98-1.08) | 0.224 | 0.92 (0.80-1.06) | 0.273 | 0.94 (0.76-1.17) | 0.596 |
| Quarter 7 | 1.06 (1.03-1.10) | <.001 | 1.03 (0.98-1.08) | 0.299 | 1.02 (0.88-1.17) | 0.798 | 1.01 (0.83-1.24) | 0.916 |
| Quarter 8 | 1.04 (1.01-1.08) | 0.018 | 1.01 (0.97-1.07) | 0.551 | 0.97 (0.84-1.11) | 0.622 | 0.93 (0.74-1.16) | 0.516 |
| ***Worsening depressive symptoms*** | |  |  |  |  |  |  |  |
| Quarter 1 | 1.08 (1.05-1.11) | <.001 | 1.13 (1.10-1.17) | <.001 | 0.91 (0.83-1.00) | 0.055 | 0.93 (0.83-1.05) | 0.233 |
| Quarter 2 | 0.97 (0.95-1.00) | 0.019 | 1.01 (0.98-1.05) | 0.368 | 0.83 (0.75-0.91) | <.001 | 0.81 (0.72-0.92) | 0.002 |
| Quarter 3 | 0.90 (0.88-0.93) | <.001 | 0.96 (0.93-1.00) | 0.064 | 0.88 (0.77-0.99) | 0.042 | 0.94 (0.80-1.11) | 0.494 |
| Quarter 4 | 0.92 (0.89-0.94) | <.001 | 0.95 (0.92-0.99) | 0.012 | 0.94 (0.83-1.07) | 0.359 | 0.93 (0.77-1.11) | 0.394 |
| Quarter 5 | 0.90 (0.87-0.93) | <.001 | 0.95(0.90-1.00) | 0.054 | 0.88 (0.74-1.06) | 0.179 | 1.00 (0.77-1.29) | 0.995 |
| Quarter 6 | 0.89 (0.86-0.93) | <.001 | 0.94 (0.89-0.99) | 0.015 | 0.81 (0.68-0.97) | 0.021 | 0.84 (0.66-1.07) | 0.153 |
| Quarter 7 | 0.90 (0.87-0.93) | <.001 | 0.95 (0.91-1.00) | 0.070 | 0.88 (0.74-1.05) | 0.170 | 0.91 (0.71-1.16) | 0.442 |
| Quarter 8 | 0.86 (0.83-0.89) | <.001 | 0.91 (0.86-0.95) | <.001 | 0.87 (0.73-1.03) | 0.112 | 0.87 (0.68-1.12) | 0.272 |

Abbreviations: ADRD, Alzheimer disease and related dementias; LTOT, long-term opioid therapy; OR, odds ratio.

^a^ Data for quarters 1 to 4 were derived from the eligible sample with ≥1 year of follow-up; data for quarters 5 to 8, with ≥2 years of follow-up.

^b^ Adjusted baseline variables (excluding baseline depressive symptoms) via the inverse probability of treatment weighting and the time-varying confounders (including use of adjuvant analgesics, use of as needed pain medications, use of any pain intervention, use of central nervous system medications, and dementia severity) as covariates.

**Figure A.1.** Schematic Diagram of Study Design, Including Time Period for Identifying Cohort Entry, Index Date for Exposure Status, Eligibility Criteria, and Outcome Follow-ups

Follow-ups

**Cohort entry**

(day 90 of the latest LTOT)

Abbreviations: NH, nursing home; ADRD, Alzheimer disease and related dementias; LTOT, long-term opioid therapy.

^1^ Eligibility criteria, unless otherwise noted, were applied during the study period from 12 months before cohort entry to the end of the 1-year and 2-year follow-ups.

^2^ Requirement of hospital stay for <60 days was applied during the period from cohort entry to the end of the 1-year and 2-year follow-ups.

^3^ Requirements for the ability to communicate and having mild or moderate dementia were applied during the 6 months before the index date.

^4^ The index date, which could occur anytime during the 1-year assessment period, was defined as the date of opioid discontinuation for LTOT discontinuers and the frequency-matched date for LTOT continuers.

1-year assessment period for LTOT

1-year assessment period for discontinuation of LTOT

**Older NH residents with ADRD**

**Index date**^4^

(for exposure status)

**Cohort re-entry**

Eligibility criteria^1^ included continuous NH stay with chronic pain but no coma, cancer, hospice, or palliative care, continuous Medicare enrollment, hospital stay(s) for ≤60 days^2^, ability to communicate^3^, and mild or moderate dementia^3^.
